# Congenital adiponectin deficiency mitigates high-fat-diet-induced obesity in gonadally intact male and female, but not in ovariectomized mice

**DOI:** 10.1038/s41598-022-21228-x

**Published:** 2022-10-05

**Authors:** Christian A. Unger, Ahmed K. Aladhami, Marion C. Hope, Sahar Pourhoseini, Mitzi Nagarkatti, Owen P. McGuinness, E. Angela Murphy, Kandy T. Velázquez, Reilly T. Enos

**Affiliations:** 1 Department of Pathology, Microbiology, and Immunology, University of South Carolina-School of Medicine, 6439 Garners Ferry Rd, Columbia, SC 29209 USA; 2 Nursing College, University of Baghdad, Baghdad, Iraq; 3 Department of Molecular Physiology and Biophysics, Vanderbilt University School of Medicine, Nashville, TN 37232 USA

**Keywords:** Metabolism, Obesity

## Abstract

Epidemiological literature indicates that women are less susceptible to type II diabetes (T2D) than males. The general consensus is that estrogen is protective, whereas its deficiency in post-menopause is associated with adiposity and impaired insulin sensitivity. However, epidemiological data suggests that males are more prone to developing T2D, and at a lower BMI, compared to females during post-menopausal years; suggesting that another factor, other than estrogen, protects females. We proposed to determine if adiponectin (APN) serves as this protective factor. An initial experiment was performed in which gonadally intact male and female mice were fed either a purified low-fat diet (LFD) or high-fat diet (HFD) (40% kcals from fat) for 16 weeks. An additional group of HFD ovariectomy (OVX) mice were included to assess estrogen deficiency’s impact on obesity. Body composition, adipose tissue inflammation, ectopic lipid accumulation as well as glucose metabolism and insulin resistance were assessed. In corroboration with previous data, estrogen deficiency (OVX) exacerbated HFD-induced obesity in female mice. However, despite a higher body fat percentage and a similar degree of hepatic and skeletal muscle lipid accumulation, female OVX HFD-fed mice exhibited enhanced insulin sensitivity relative to HFD-fed males. Therefore, a subsequent HFD experiment was performed utilizing male and female (both gonadally intact and OVX) APN deficient mice (APN^−/−^) and wildtype littermates to determine if APN is the factor which protects OVX females from the similar degree of metabolic dysfunction as males in the setting of obesity. Indirect calorimetry was used to determine observed phenotype differences. APN deficiency limited adiposity and mitigated HFD-induced insulin resistance and adipose tissue inflammation in gonadally intact male and female, but not in OVX mice. Using indirect calorimetry, we uncovered that slight, but non-statistically significant differences in food intake and energy expenditure leading to a net difference in energy balance likely explain the reduced body weight exhibited by male APN-deficient mice. In conclusion, congenital APN deficiency is protective against obesity development in gonadally intact mice, however, in the setting of estrogen deficiency (OVX) this is not true. These findings suggest that gonadal status dictates the protective effects of congenital APN deficiency in the setting of HFD-induced obesity.

## Introduction

Epidemiological literature indicates that women are less susceptible to type II diabetes (T2D) than males. This is largely regulated by sex hormones—estrogen, in particular. The general consensus is that estrogen is protective, whereas its deficiency in post-menopausal women is associated with adiposity and impaired insulin sensitivity^1–3^. However, epidemiological data suggests that males are more prone to developing T2D, and at a lower BMI, as compared to females during mid-life (post-menopausal, estrogen-deficient years); suggesting that another factor, other than estrogen, protects females^4–6^. Human data suggests that adiponectin (APN) increases during estrogen deficiency and may serve as this protective factor^7^, but this has not been mechanistically examined.

APN is a well-established adipokine whose role as a beneficial or detrimental factor in a variety of diseases remains controversial^8–11^. Although the general consensus is that APN has a beneficial effect on metabolic processes, contrary publications suggest that APN deficiency actually reduces body fat%, protects against NAFLD development, and enhances skeletal muscle beta oxidation^12–14^. Furthermore, a limitation of previous investigations is that the primary focus in these experiments has been on male mice. This is surprising given that both in vitro and epidemiological investigations suggest estrogen status plays a significant role in impacting APN production. For example, experiments performed in adipocytes show that estrogen reduces intra- and extra-cellular APN^15^. In humans, there are strong negative associations of serum APN with estrogen and the stage of menopause^7,16^. Additionally, estrogen replacement therapy in post-menopausal females reduces plasma APN levels^17^. To date the in vivo interaction of APN and estrogen status in the setting of diet-induced obesity has not been investigated.

Here we show that ovariectomized female mice on a high-fat diet (HFD) present a similar degree of visceral adiposity, a greater body fat percentage, and an analogous degree of hepatic steatosis as HFD-fed male mice, yet present a significantly superior insulin sensitive phenotype. In this investigation, we probe to answer the question whether APN is a key molecule that regulates this enhanced metabolic phenotype in an estrogen-deficient state relative to similarly obese males.

## Material and methods

### Animals

For experiments in which APN KO were not utilized, wildtype (WT) mice on a C57BL/6J background were purchased from Jackson Laboratories (Bar Harbor, ME). For all other experiments utilizing APN KO mice, APN^+/-^ mice on a C57BL/6 background (a kind gift from Dr. Philipp Scherer, UT-Southwestern) were bred in order to produce APN KO and WT littermate controls. Mice were genotyped utilizing the following primers: ApnWTf: 5′-TTGGACCCCTGAACTTGCTTCACACC-3′, ApnWTr: 5′-TCCTGAGTTCAATTCCCAGCACCCAC-3′, ApnKOr2: 5′-GGATGCGGTGGGCTCTATGGCTTC-3′ with the following PCR conditions: (95 °C, 5 min)—[(95 °C, 15 s)—(62 °C, 30 s)—(72 °C, 30 s)] X 35—(72 °C, 3 min). All ovariectomy surgeries were performed at ≈ 10 weeks of age when female mice were sexually mature. Mice were given one week recovery before the initiation of dietary treatment. The sample size for each experimental group is presented in the legend of each figure. All methods were in accordance with the American Association for Laboratory Animal Science, and the Institutional Animal Care and Usage Committee of the University of South Carolina approved all experiments. The authors complied with the ARRIVE guidelines.

### Diets

At 11 weeks of age, mice were assigned to receive either a purified low-fat diet (LFD) or a custom high-fat diet for a duration of 16 weeks. We have found this length of feeding to be sufficient to induce an obese phenotype characterized by impaired glucose metabolism, hyperinsulinemia, and adipose tissue inflammation^18–20^. The LFD utilized in this experiment was the open-source, purified AIN-76A diet (3.77 kcal/g) comprised of 69%, 12%, and 19% of total kcals from carbohydrate, fat, and protein, respectively. The HFD (4.57 kcal/g) was a purified diet comprised of 47%, 40%, and 13% of total calories from carbohydrate, fat, and protein, respectively, with saturated fat making up 12% of total calories in order to mimic the standard American diet (BioServ, Frenchtown, NJ). Details and previous use of this diet are provided elsewhere^18–26^. Mice were housed, 3–5/cage, maintained on a 12:12-h light–dark cycle in a low stress environment (22 °C, 50% humidity, low noise) and given food and water ad libitum. All methods were in accordance with the American Association for Laboratory Animal Science, and the Institutional Animal Care and Usage Committee of the University of South Carolina approved all experiments.

### Body weight and body composition

Body weight was monitored on a weekly basis throughout the study. Body composition was assessed after ≈ 12 and ≈ 16 weeks of diet in order to use lean mass as the basis for the dose of glucose and insulin administration for glucose and insulin tolerance tests, respectively. The intention for assessing metabolic outcomes at two different time points was to determine if the length of dietary feeding differentially impacted metabolic outcomes between groups. For this procedure, mice were briefly anesthetized via isoflurane inhalation, and lean mass, fat mass, and percent body fat were assessed by dual-energy X-ray absorptiometry (Lunar PIXImus).

### Metabolism

Fasting blood glucose and insulin levels were assessed after ≈ 12 and 16 weeks of dietary treatment. After a 5-h fast, blood samples were collected from the tip of the tail. A glucometer (Bayer Contour, Mishawaka, IN) was used to determine blood glucose concentrations in whole blood. Collected blood was centrifuged at 4000 rpm for 10 min at 4 °C. Plasma insulin concentrations were analyzed according to the manufacturer’s instructions using a mouse insulin ELISA kit (Mercodia, Winston Salem, NC). Glucose and insulin tolerances tests (GTTs and ITTs, respectively) were performed after ≈ 12 and ≈ 16 weeks of dietary treatment. GTTs and ITTs were performed a week apart (e.g. after 11.5 and 12.5 weeks and after 15.5 and 16.5 weeks of dietary treatment) to allow for animal recovery between metabolic tests. For these procedures, mice were fasted for 5 h, and glucose or insulin was administered intraperitoneally at 2 g/kg lean mass or 0.75 U/kg lean mass, respectively. A glucometer (Bayer Contour) was used to measure blood glucose concentrations (tail sampling) intermittently over a 2-h period (0, 15, 30, 60, 90, and 120 min) for GTTs and intermittently over a 45-min period (0, 15, 30, and 45 min) for ITTs. Area of the Curve (AUC) after subtracting baseline blood glucose levels was calculated using the trapezoidal rule based off of the insights by Virtue and Vidal-Puig^27^. Blood was collected from the tip of the tail during the GTT (0, 15, 30, 60, and 120 min) in order to assess the insulin response to the GTT for a subset of mice from each group. Fasting serum was collected (using non-heparinized capillary tubes) for free fatty acid (FFA) analysis using a commercially available kit (Wako Diagnostics, Richmond, VA). Fasting plasma was collected for circulating triglyceride levels using a commercially available kit (Infinity Triglyceride Reagent, Thermo Fisher Scientific, Waltham, MA). In order to assess tissue-specific insulin signaling, a sub-set of mice were fasted for 5 h and were subsequently administered 0.75 U/kg lean mass of insulin or PBS. At 7 min after insulin administration, the mice were euthanized (cervical dislocation) and the gastrocnemius was quickly harvested and snap-frozen in liquid nitrogen in order to assess insulin signaling in the gastrocnemius skeletal muscle.

### Tissue collection

After 16 weeks of dietary treatment, mice were euthanized via isoflurane inhalation for tissue collection. Epididymal, mesenteric, and perirenal fat pads, as well as the liver, and skeletal muscle (gastrocnemius) were removed, weighed, and immediately snap-frozen in liquid nitrogen and stored at − 80 °C or fixed in 4% formalin until analysis.

### Hepatic and skeletal muscle lipid content

Hepatic and skeletal muscle triglyceride assessments were performed as previously described^28^. For total hepatic lipid assessment, lipids were isolated from the liver utilizing a modified folch extraction method and quantified gravimetrically, as previously described^20,26^.

### Liver histology

Formalin-fixed tissues were embedded in paraffin blocks and sectioned (Instrumentation Resource Facility at the University of South Carolina). Livers were stained with hematoxylin and eosin (Biocare, Pacheco, CA). Representative images were taken at 10x (Nikon E600).

### Plasma leptin and high-molecular weight (HMW) APN assessment

Plasma leptin and HMW APN were determined using commercially-available ELISA kits (R&D Systems, Minneapolis, MN and ALPCO, Salem, New Hampshire for leptin and HMW APN, respectively).

### Quantitative real-time PCR

An E.Z.N.A. Total RNA Kit (Omega Bio-Tek, Norcross, GA) was used to isolate RNA from gonadal adipose tissue. Taqman reverse transcription reagents and probe assays (Applied Biosystems, Waltham, MA) were used to reverse transcribe and analyze expression of the following genes in gonadal adipose tissue: *F4/80, CD11c, CD206, MCP-1, TNFα, IL-10, CXCL14, TLR2, PPARγ, and PGC1-α*. Potential reference genes (*HPRT, 18 s, GAPDH, β-Actin, HMBS, TBP, H2AFV, and B2M*) were analyzed for stability using Qbase + software (Biogazelle, Ghent, Belgium). The optimal number of reference genes was determined by Qbase + (*HMBS & H2afv*), and the geometric mean of these genes was used as the normalization factor for each analysis. Gene expression was quantified using the ΔΔC_T_ method and Qbase + software^29^.

### Western blotting

Briefly, gonadal fat and skeletal muscle were homogenized in Mueller Buffer containing a protease inhibitor cocktail (Sigma Aldrich, St. Louis, MO)^30^. Total protein concentrations were determined by the Bradford method. Equal amounts of crude protein homogenates were fractioned on hand-casted 12% SDS–polyacrylamide gel and electrophoretically transferred to a PVDF membrane using a Genie Blotter (IDEA Scientific, Minneapolis, MN). 0.5 µL of plasma was used to verify the absence of circulating adiponectin in APN KO mice. Membranes were stained with a Ponceau S solution in order to verify equal protein loading and transfer efficiency. Subsequently, membranes were blocked for 1 h in 5% milk in Tris-buffered saline, 0.1% Tween-20 (TBST). The AKT, p-AKT^Ser473^, and p-JNK antibodies (Cell Signaling, Danvers, MA) as well as the adiponectin antibody (Biovendor, Karasek, Czech Republic) (were diluted 1:1000 in a 5% milk-TBST overnight at 4ºC and JNK (Cell Signaling, Danvers, MA for 2 h at room temperature). An anti-rabbit (Cell Signaling, Danvers, MA) IgG horseradish peroxidase conjugated secondary antibody was diluted 1:2000 in 5% milk-TBST and incubated for 1 h at room temperature. An enhanced chemiluminescent substrate for detection of horseradish peroxidase (Thermo Scientific, Watham, MA) was used to visualize the antibody-antigen interaction. Autoradiography films were scanned and blots were quantified using scientific imaging software (Image J). After completion of the western blot, all membranes were stained with Amido black or ponceau^18,23,31^, and the densitometry of each lane was calculated using Quantity One Software (Biorad, Hercules, CA) allowing for total protein normalization. This method of normalization has been shown to be more accurate than typically used loading controls^32^. Complete western blots are presented in Supplementary Fig. 8. Please note as western blots were captured with autoradiography film, the films were pre-cut or cut after film developing prior to scanning for imaging.

### Food intake assessment

In experiments in which food individual food intake was assessed, mice were singly-housed for 16 weeks. Individual food intake for each mouse was assessed on a weekly basis.

### Indirect calorimetry and behavioral phenotyping

Male APN KO and WT littermate controls (n = 7–8/group) were fed a HFD for 6 weeks, underwent body composition analysis (DEXA), and were placed into a 16-cage Promethion multichannel continuous measurement indirect calorimetry system (Sable System International, Las Vegas, NV, USA) on a 12-h light and 12-h dark cycle for a 11-day period. After a 1-day acclimation period, food intake, water consumption, body mass, total activity, total energy expenditure, resting energy expenditure, oxygen consumption, carbon dioxide production, respiratory quotient, and animal locomotion (all meters) were determined and analyzed over a ten-day period^33^. All data (besides RER and all meters = two-tailed student’s T-test) were analyzed using an ANCOVA with lean mass as a covariate utilizing the MMPC Statistical Analysis Page (https://www.mmpc.org/shared/regression.aspx). CalR was used to assess energy balance and for the creation of the net energy balance and RER figures over time^34^.

### Statistical analysis

Data were analyzed using commercially available statistical software: Prism 9 (GraphPad Software, La Jolla, CA, www.graphpad.com). A one-way ANOVA followed by a Newman-Keuls post hoc test was used for all experiments utilizing WT mice only. A two-way ANOVA (genotype vs. sex) followed by a Newman-Keuls post-hoc analysis was used to assess differences between Female WT, Female APN KO, Male WT, and Male APN KO mice as well as a two-way ANOVA (genotype vs. gonadal status) followed by a Newman-Keuls post-hoc analysis in order to determine differences between Male WT, Male APN KO, OVX WT, and OVX APN KO. A two-tailed student’s t-test was used when only two groups were compared. Any statistical test that did not pass the equal-variance test (Bartlett’s test for equal variances) was log-transformed and then reanalyzed. Data are presented as means ± SE, and the level of significance was set at *P* < 0.05.

## Results

### Despite similar increases in adiposity as a result of HFD consumption, OVX mice exhibit reduced adipose tissue inflammation compared to HFD-fed males

**Figure 1:**
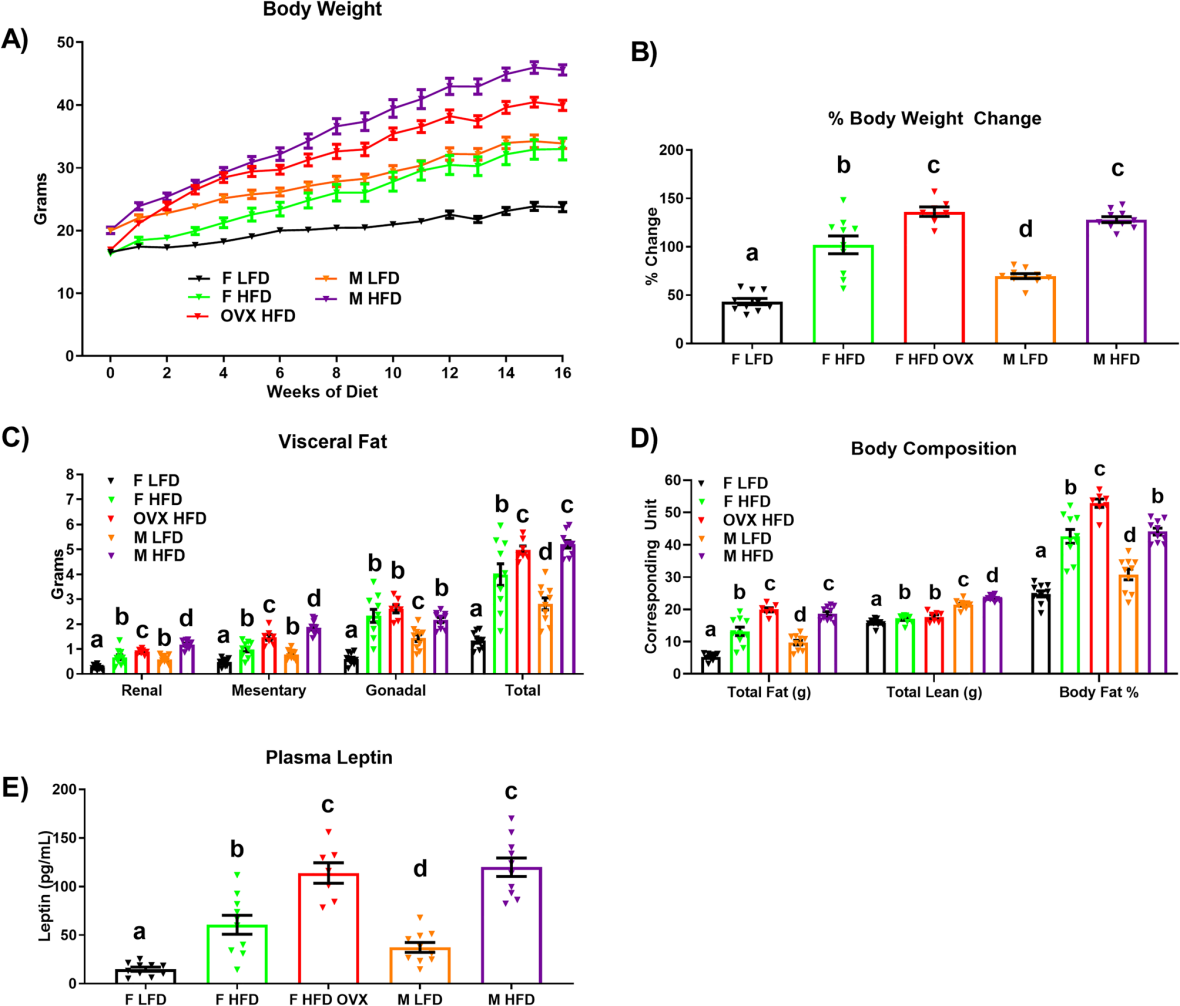
Female estrogen deficiency leads to a similar degree of adiposity as males in the setting of a high-fat diet (HFD). Male and female mice were fed a low-fat diet (LFD) or HFD for 16 weeks (n = 10/group). An additional group of HFD OVX mice (n = 7) were included in the study. (**A**) Body weight over the course of the study, (**B**) % change in body weight from baseline, (**C**) visceral fat accumulation, (**D**) body composition, and (**E**) plasma leptin levels. Data is presented as mean ± SE. Bar graphs not sharing a common letter are significantly different from one another (P < 0.05).

**Figure 2:**
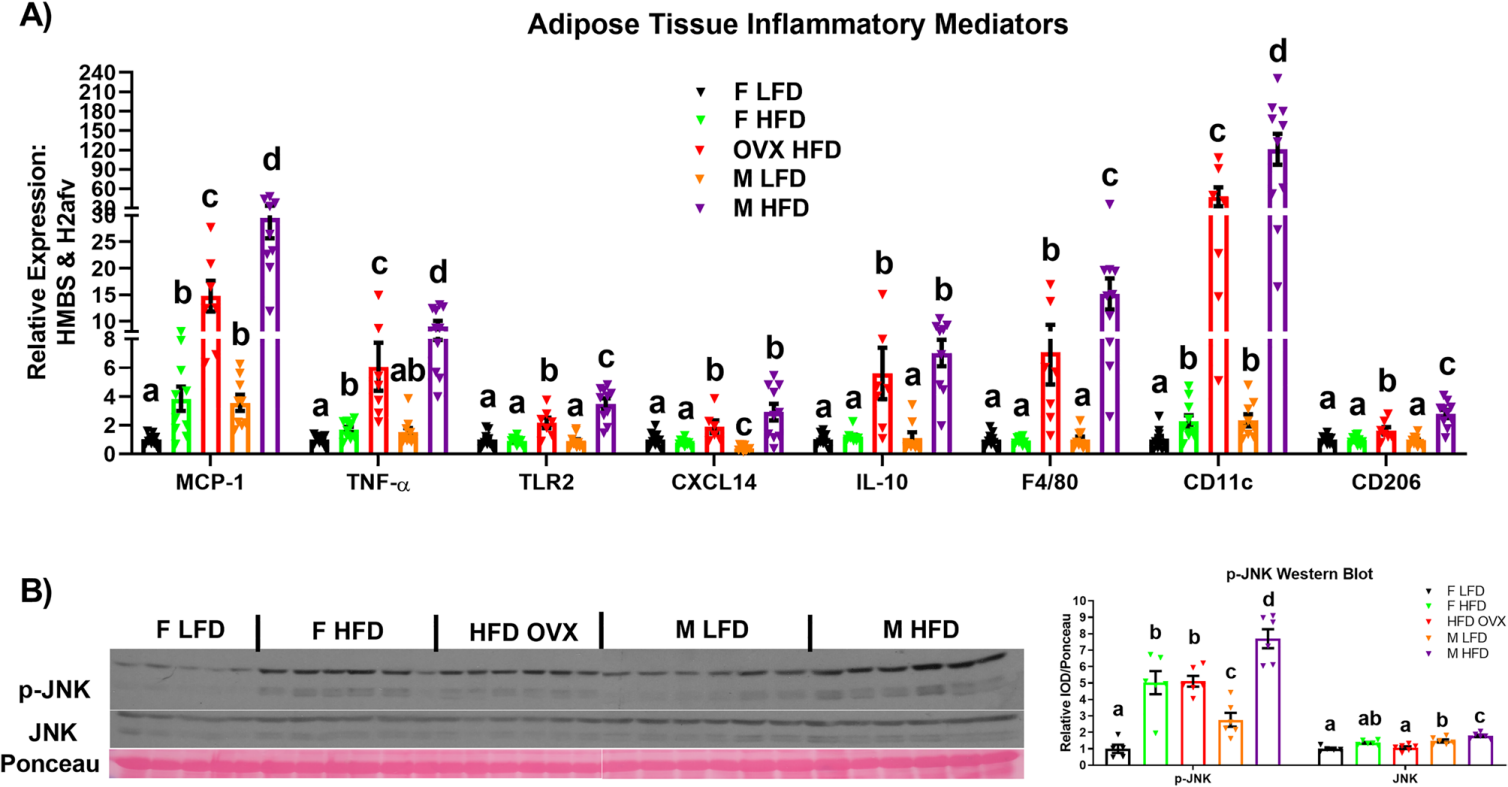
Despite a similar degree of adiposity, HFD estrogen-deficient females exhibit reduced adipose tissue inflammation compared to HFD males. After 16 weeks of LFD or HFD, adipose tissue inflammation was assessed via (**A**) qRT-PCR for inflammatory markers (n = 7–10), and (**B**) via western blot for JNK activation (n = 5–6). Data is presented as mean ± SE. Bar graphs not sharing a common letter are significantly different from one another (P < 0.05).

Male and female mice were fed a LFD or HFD for 16 weeks (n = 10/group). An additional group of HFD OVX mice (n = 7) were included in the study design in order to assess the impact of estrogen deficiency on the development of the obese phenotype. Body weight gain, as well as visceral and total fat accumulation, and plasma leptin were greatest in the male and OVX HFD mice, followed by the female HFD, male LFD, and finally female LFD mice (Fig. 1A–E) (P < 0.05). However, because male mice displayed greater lean mass than female mice, the body fat% was greatest in the OVX HFD mice compared to all other groups (Fig. 1D) (P < 0.05). In order to determine the impact that weight gain had on adipose inflammation, we performed qRT-PCR in the gonadal adipose tissue for all experimental groups (Fig. 2A). Within males, HFD feeding increased the gene expression of all inflammatory mediators: *MCP-1, TNF-α, TLR2, CXCL14, IL-10* and macrophage markers measured: *F4/80* (general macrophage marker), *CD11c* (M1 macrophage marker), and *CD206* (M2 macrophage marker) (P < 0.05). As it has already been established that a sexual dichotomy exists with respect to the inflammatory response to HFD consumption between males and females^35^, it was not surprising to find that HFD-fed females only displayed increased gonadal *MCP-1, TNF-α,* and *CD11c* gene expression relative to LFD-fed mice (P < 0.05), but not *TLR2, CXCL14, IL-10, F4/80,* and *CD206* mRNA content. Given that estrogen is known to play an important role in regulating inflammatory and metabolic processes^36,37^, it was anticipated and confirmed that estrogen deficiency in a HFD setting increased the gene expression of all inflammatory markers measured relative to intact female HFD mice (P < 0.05). Nonetheless, it was unexpected to find that despite the similar levels of gonadal fat, male HFD mice displayed significant increases in gonadal adipose tissue inflammation at the mRNA level (*MCP-1, TNF-α, TLR2, F4/80, CD11c, and CD206*), and at the protein level as evidenced by increased JNK phosphorylation protein content (Fig. 2B) (P < 0.05).

### Despite similar increases in adiposity and ectopic lipid accumulation as a result of HFD consumption, OVX mice exhibit enhanced insulin sensitivity relative to HFD-fed males

**Figure 3:**
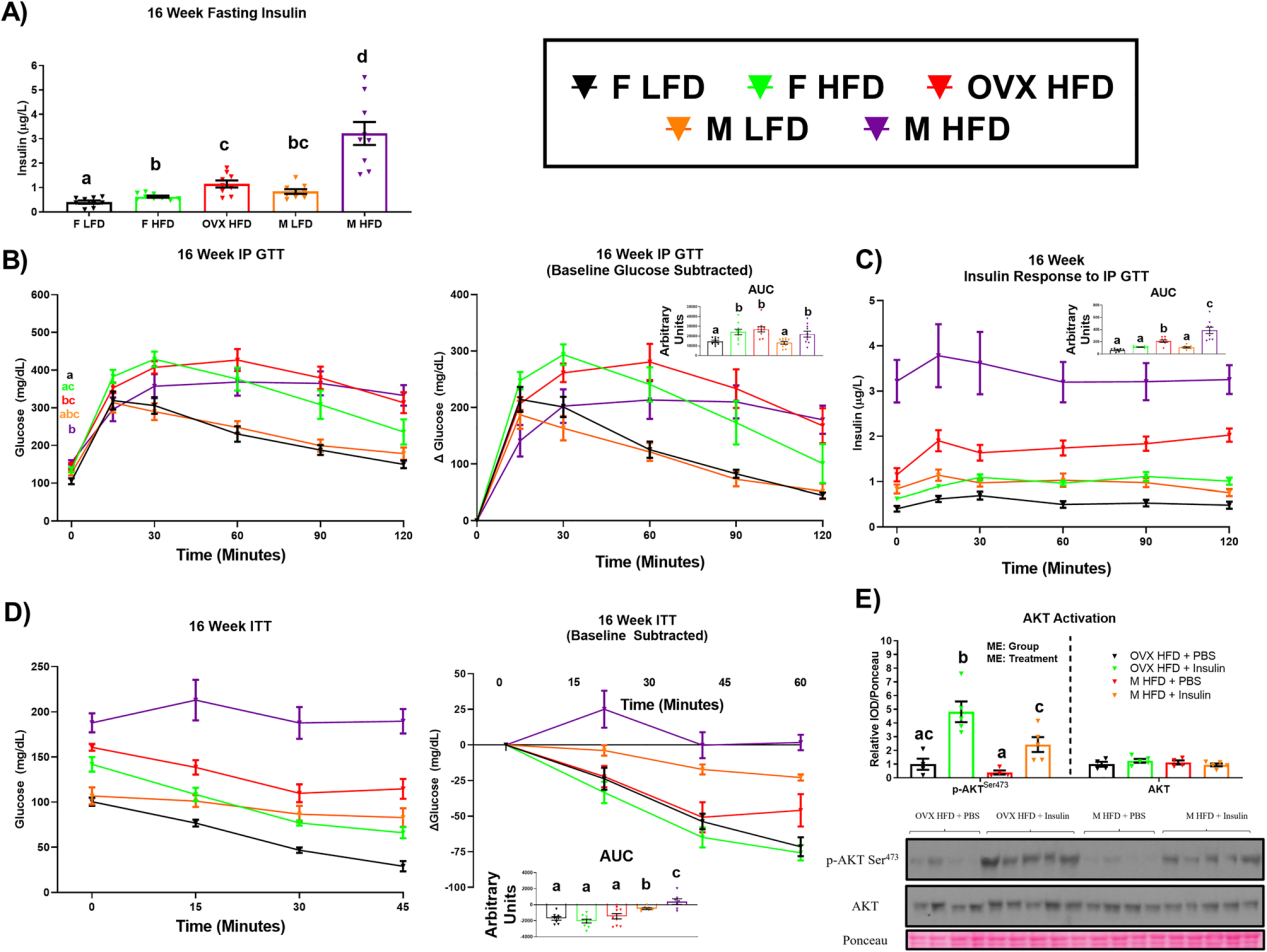
Despite a similar degree of adiposity, HFD estrogen-deficient females exhibit enhanced insulin sensitivity relative to HFD males. After 16 weeks of LFD or HFD, mice (n = 10) were assessed metabolically by examining (**A**) fasting insulin levels, (**B**) a glucose tolerance test (GTT), (**C**) insulin response to the GTT, (**D**) insulin tolerance test (ITT), and (**E**) skeletal-muscle-specific insulin-induced AKT activation (n = 4–5). Data is presented as mean ± SE. Bar graphs not sharing a common letter are significantly different from one another (P < 0.05).

In our initial preliminary study, we assessed fasting blood glucose incrementally throughout the experimental protocol as well as fasting insulin after 16 weeks of diet consumption (Supplementary Fig. 1). All groups displayed significantly higher fasting blood glucose and insulin levels compared to female LFD mice (P < 0.05). However, the male HFD mice displayed a 1.25 × and 3.4 × increase in fasting blood glucose AUC and insulin levels, respectively, relative to OVX HFD mice (Supplementary Fig. 1) (P < 0.05). In order to perform more advanced metabolic tests to corroborate our initial findings, a second study was performed (n = 10/group) in which metabolic outcomes were assessed after 12 and 16 weeks of dietary feeding. After 12 weeks of HFD consumption, our initial results were corroborated—despite presenting a lower body fat% (Supplementary Fig. 2A), the male HFD mice displayed significantly elevated fasting insulin and glucose levels compared to OVX HFD mice (Supplementary Fig. 2B, C) (P < 0.05). However, although we did not see a difference in the AUC for the GTT between the OVX and male HFD mice, the OVX mice did display lower fasting blood glucose levels 90 and 120 min into the GTT. Additionally, the OVX HFD mice were significantly more insulin sensitive than the male HFD mice as evidenced by an ITT (Supplementary Fig. 2C) (P < 0.05). After 16 weeks of diet, GTTs and ITTs were performed yet again (Fig. 3). Male HFD mice continued to display elevated fasting insulin levels compared to OVX HFD mice (Fig. 3A) (P < 0.05). However, no difference was found in the AUC of the GTT between the male HFD and OVX HFD mice, nor did the OVX HFD mice display lower glucose levels at 90 and 120 min into the GTT after 16 weeks of feeding as was previously seen after 12 weeks of feeding (Fig. 3B). Interestingly, however, the OVX HFD mice displayed significantly lower circulating insulin over the course of the GTT, suggesting their enhanced insulin sensitivity relative to the male HFD mice (Fig. 3C). The 16-week ITT corroborated our initial 12-week ITT finding, in that the OVX HFD mice displayed enhanced insulin sensitivity relative to the male HFD mice (Fig. 3D) (P < 0.05). Additionally, we examined skeletal muscle AKT activation after an insulin bolus and found increased AKT activation in the skeletal muscle of OVX HFD mice relative to male HFD mice (Fig. 3E) (P < 0.05). In order to determine if ectopic lipid accumulation may be responsible for the differences in insulin sensitivity between the male and OVX HFD mice we examined hepatic and skeletal muscle triglyceride accumulation (Supplementary Fig. 3A-B). No difference was found in triglyceride accumulation in these two metabolic tissues between the OVX HFD and male HFD mice.

### Circulating high-molecular weight (HMW) adiponectin (APN) is increased in OVX HFD mice

**Figure 4:**
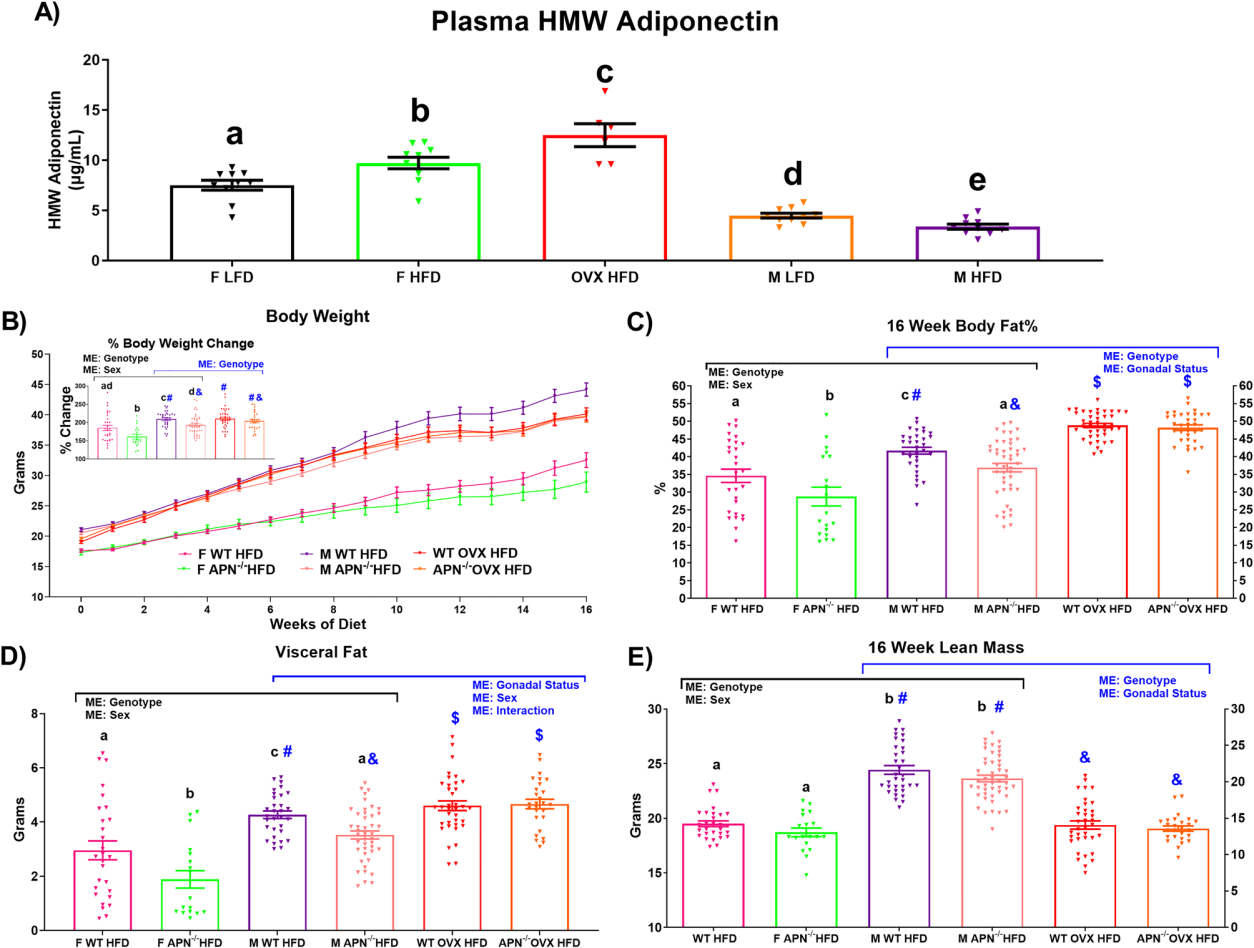
Estrogen deficiency increases high-molecular-weight (HMW) adiponectin (APN), and APN deletion mitigates HFD-induced body fat accrual in gonadally intact males and females but not in ovariectomized females. After 16 weeks of LFD or HFD, (**A**) plasma was assessed for HMW APN in WT mice (n = 6–10). A subsequent 16-week HFD experiment was performed utilizing male and female (both intact and OVX) APN deficient mice (M and F APN^−/−^) and wildtype (WT) littermate controls (n = 19–46). The data is presented as follows, (**B**) body weight over the course of the study as well as % body weight change from baseline, (**C**) body fat %, (**D**) visceral fat accumulation, and (**E**) lean mass. Bar graphs not sharing a common letter or symbol are significantly different from one another (P < 0.05). ME = Main Effect.

Our data suggest that, despite similar levels of visceral adiposity and ectopic lipid accumulation, estrogen-deficient OVX mice are still protected against the same degree of adipose tissue inflammation and metabolic dysfunction exhibited by male HFD mice. Our goal was to determine the potential mediator(s) of these effects. Subsequently, we performed an ELISA to examine circulating HMW APN as the HMW form of APN has been shown to possess insulin sensitizing properties, and to be elevated in females relative to males^15,38^. Of further interest is the fact that estrogen has been shown to be a negative regulator of APN^15^. Taking this into consideration, it was not surprising that OVX HFD mice displayed the highest levels of circulating HMW APN followed by the F HFD, F LFD, M LFD, and M HFD mice (Fig. 4A) (P < 0.05). Based on this finding, we designed our subsequent experiments to test the hypothesis that APN increases following estrogen deficiency, which serves as a protective factor against impaired insulin signaling and adipose inflammation exhibited by male mice of similar body composition.

### APN deficiency mitigates HFD-induced body fat accrual in gonadally intact males and females but not in OVX females

WT and APN KO male and female (both gonadally intact and OVX) mice were placed on a HFD for 16 weeks (n = 19–46). Prior to the initiation of any experiment, in addition to genotyping, we confirmed via western blot that all APN KO mice were APN deficient (Supplementary Fig. 4). When comparing gonadally intact males and females, there was a main effect for APN deficiency to decrease body weight gain, body fat%, visceral fat accumulation, and lean mass (Fig. 4B–D) (P < 0.05). Post-hoc analysis showed that female and male APN KO mice displayed less body weight gain, body fat%, and visceral fat mass, but not lean mass compared to their WT littermate controls (P < 0.05). When comparing male and OVX mice, a main effect of genotype was found for body weight gain, body fat%, visceral fat accumulation and lean mass as APN deficiency decreased all of these outcomes (P < 0.05). A main effect of gonadal status was also found with respect to body fat% and visceral fat with OVX increasing these parameters as well as for lean mass with OVX decreasing this outcome (P < 0.05). With respect to post-hoc analyses, male APN KO mice displayed a lower body weight gain, body fat%, and visceral fat compared to all other groups, whereas OVX WT and OVX APN KO mice displayed an increased body fat%, visceral fat accumulation, and less lean mass than both male groups (P < 0.05).

### APN deficiency enhances insulin sensitivity in males and females, but has no beneficial metabolic effects in OVX females

Fasting blood glucose and insulin were assessed at 12 and 16 weeks of dietary treatment as well as fasting serum free-fatty acids and plasma triglycerides after 16 weeks of dietary treatment (Supplementary Fig. 5). Among gonadally intact males and females there was a main effect of sex to impact blood glucose levels as male mice displayed higher fasting blood glucose levels after 12 and 16 weeks of dietary treatment compared to female mice (Supplementary Fig. 5A-B) (P < 0.05). Post-hoc analysis showed that male APN KO mice displayed significantly lower fasting blood glucose levels after 12 and 16 weeks of dietary treatment compared to WT littermate controls (P < 0.05). When comparing male and OVX mice, a main effect for gonadal status was found at 12 weeks for fasting blood glucose with OVX mice presenting lower blood glucose concentrations than male mice (P < 0.05). With respect to insulin, when comparing gonadally intact males and females, main effects for both sex and genotype were found after 12 and 16 weeks of dietary treatment with females presenting lower insulin levels than males and APN KO mice presenting lower insulin levels than WT mice (Supplementary Fig. 5C-D) (P < 0.05). Post-hoc analysis showed that male APN KO mice presented lower fasting insulin levels both at 12 and 16 weeks of dietary treatment compared to WT male mice, whereas female APN KO mice only presented lower insulin levels after 16 weeks of dietary treatment compared to female WT mice (P < 0.05). When comparing male and OVX mice with respect to insulin levels, there were significant main effects for genotype (APN KO↓) and gonadal status (OVX↓) after 12 and 16 weeks of dietary treatment and an interaction (male APN KO↓ vs. male WT only) after 12 weeks of dietary treatment (Supplementary Fig. 5C-D) (P < 0.05). Regarding free-fatty acid and triglyceride levels, only a main effect of sex was found to be statistically significant with female mice exhibiting lower levels of these molecules compared to male mice (Supplementary Fig. 5E-F) (P < 0.05). On the other hand, when comparing male and OVX mice, a main effect was found for gonadal status with OVX mice displaying higher values for both lipid molecules relative to male mice (P < 0.05).

**Figure 5:**
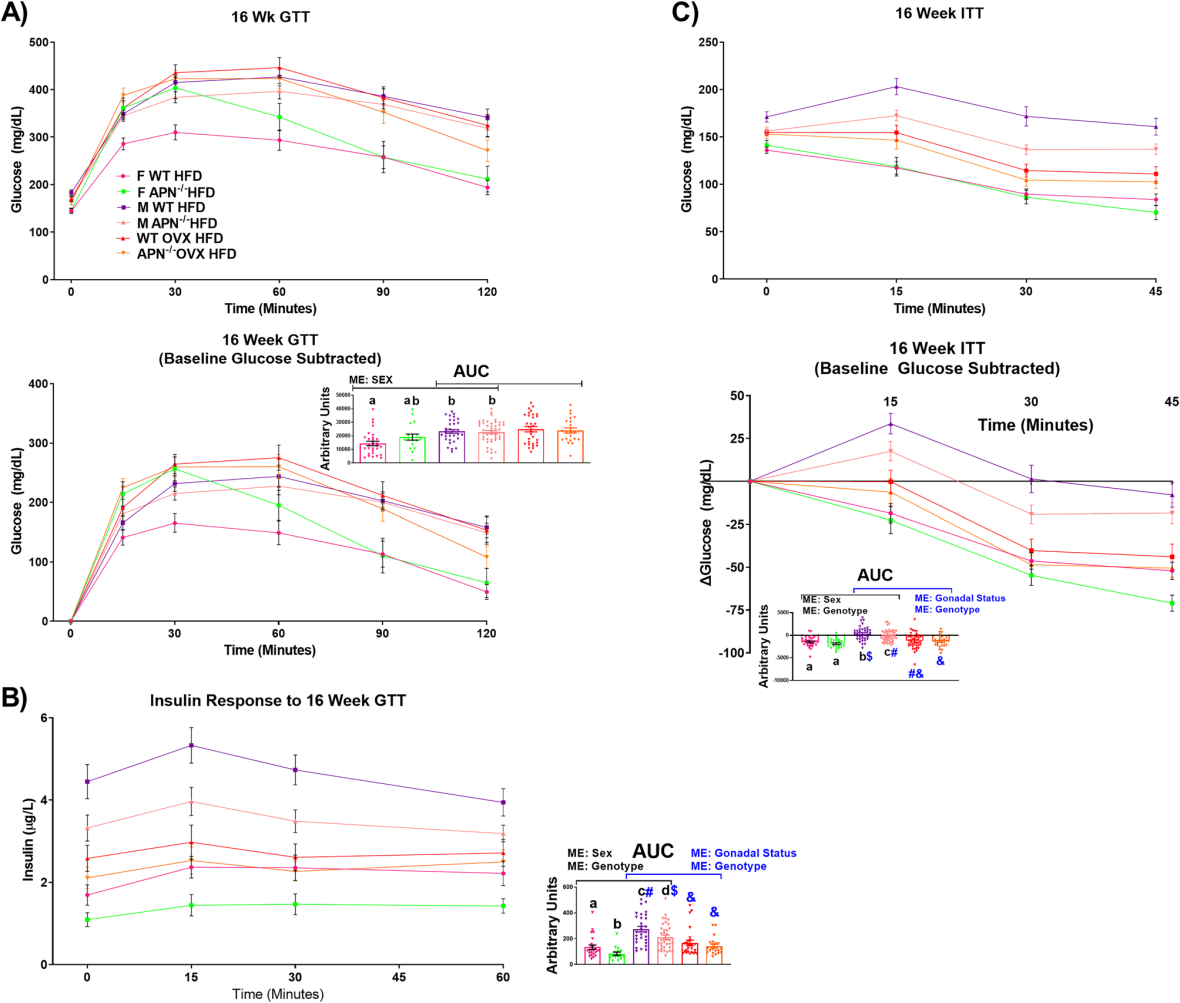
Adiponectin deficiency enhances insulin sensitivity in males and females, but has no beneficial metabolic effects in ovariectomized females. After 16 weeks of HFD, mice (n = 19–46) were assessed metabolically by examining a (**A**) GTT, (**B**) insulin response to the GTT, and (**C**) ITT. Data is presented as mean ± SE. Bar graphs not sharing a common letter or symbol are significantly different from one another (P < 0.05). ME = Main Effect.

In addition to assessing fasting metabolic parameters, we also performed metabolic tests (GTTs and ITTs) after 16 weeks of dietary treatment (Fig. 5). With respect to the GTT, when comparing males and females, a main effect of sex was found to be statistically significant with males presenting impaired glucose tolerance relative to females (Fig. 5A) (P < 0.05). No statistically significant main effects with respect to the GTTs were found between male and OVX mice. Interestingly, however, we analyzed blood insulin concentrations over the course of the GTT and found significant main effects of both sex (females < insulin AUC) and genotype (APN KO < insulin AUC) to impact blood insulin concentrations (Fig. 5B) (P < 0.05). The post-hoc analysis revealed that both male and female APN KO presented significantly lower insulin levels over the course of the GTT than WT mice, suggesting that the APN KO mice exhibit greater insulin sensitivity than their WT littermates given that it took less insulin to elicit the same overall glucose response during the GTT (P < 0.05). When comparing male and OVX mice, significant main effects for gonadal status (OVX mice < insulin AUC) and genotype (APN KO < insulin AUC) were observed. However, the post-hoc analysis revealed no significant difference between WT OVX and APN OVX mice.

With respect to the ITT, APN deficiency was found to be beneficial with respect to insulin sensitivity as a main effect of genotype was evident for all 2-way ANOVA comparisons (Fig. 5C) (P < 0.05). Specifically, post-hoc analysis revealed that male APN KO mice were more insulin sensitive than male WT mice, however, this was not true when comparing female WT and APN KO mice and OVX WT and APN KO mice (P < 0.05). It should be noted, however, that the inability to detect an AUC difference between the female WT and female APN groups may be explained by the fact that both groups of mice presented enhanced insulin sensitivity compared to their male counterparts. The ITT is not a delicate enough test to tease out differences in insulin sensitivity between highly insulin sensitive mice. Thus, given the fact that the female APN KO mice presented with lower fasting insulin levels as well as lower insulin levels over the course of the GTT with a similar net glucose response, it is suggested that the female APN KO mice present enhanced insulin sensitivity compared to their female WT controls.

### APN deficiency limits adipose tissue inflammation in gonadally intact males and females, but not OVX mice

**Figure 6:**
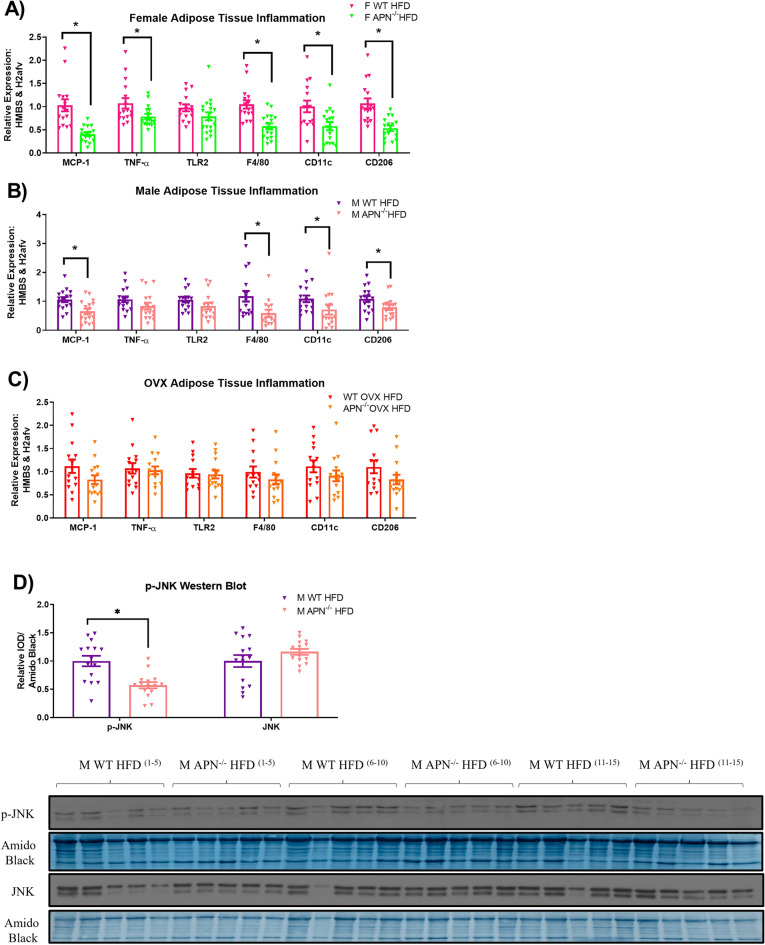
Adiponectin deficiency limits adipose tissue inflammation in gonadally intact males and females, but not ovariectomized females. After 16 weeks of HFD, adipose tissue inflammation was assessed via qRT-PCR in (**A**) F WT and APN^−/−^ mice (n = 15–16), (**B**) M WT and APN^−/−^ mice (n = 15–16), and (**C**) OVX WT and APN^−/−^ mice (n = 15–16), as well as (**D**) western blot analysis of p-JNK activation in M WT and APN^−/−^ mice (n = 15). Data is presented as mean ± SE. *Signifies statistically significant differences (P < 0.05).

Adipose tissue inflammation was assessed via qRT-PCR and is presented in Fig. 6. In general, adiponectin deficiency reduced inflammatory markers—macrophage markers, in particular—in both gonadally intact male and female mice (Fig. 6A,B) (P < 0.05). Interestingly, however, APN deficiency did not impact adipose tissue inflammation in the OVX mice (Fig. 6C). In order to corroborate the decrease in inflammation we observed at the mRNA level, we performed a western blot for p-JNK in the adipose tissue of male APN KO and WT mice (Fig. 6D). As expected, we found JNK activation to be significantly lower in the male APN KO mice (P < 0.05).

**Figure 7:**
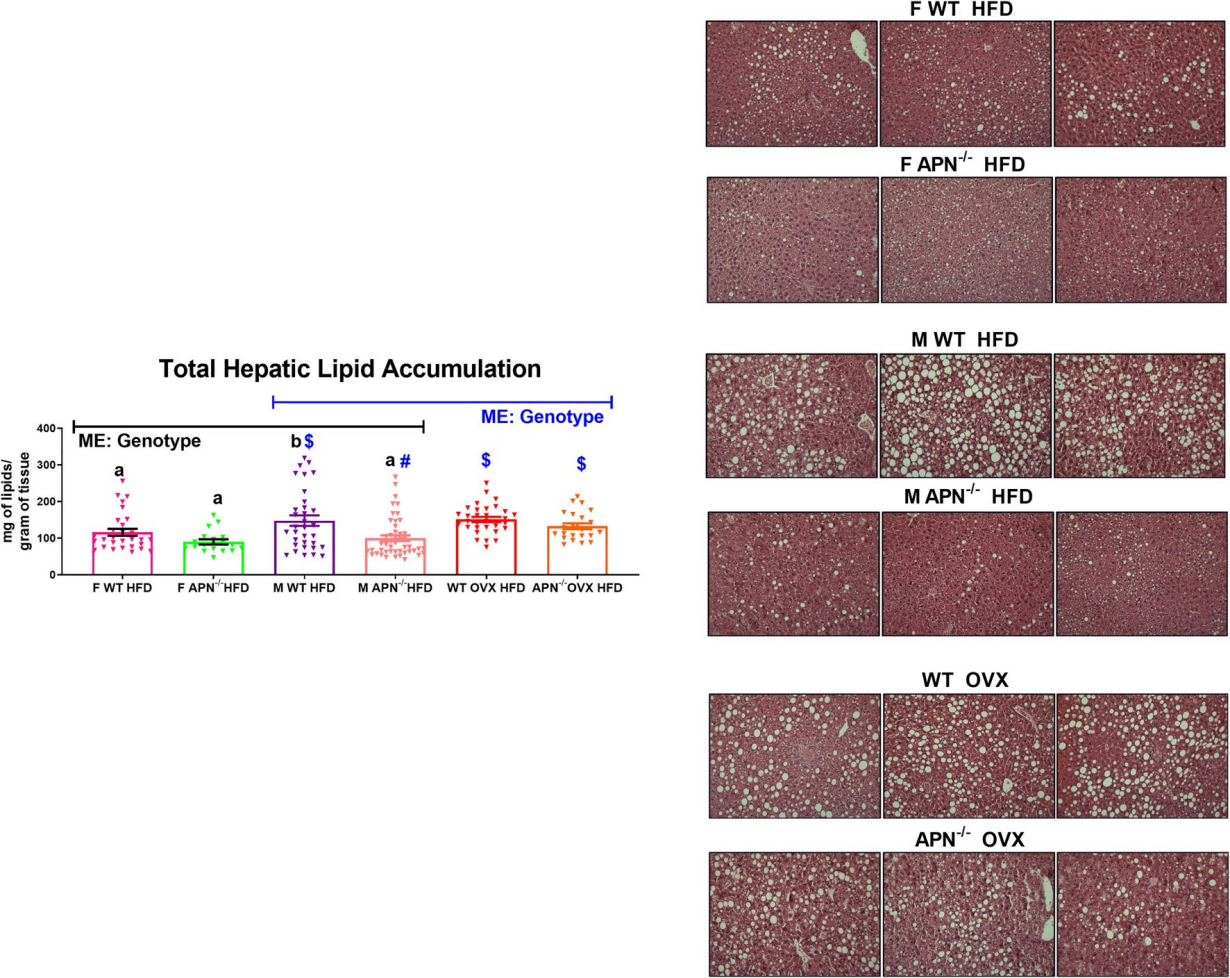
Adiponectin deficiency dramatically reduces hepatic steatosis in male mice. After 16 weeks of HFD, the liver was assessed for hepatic lipid accumulation and was histologically examined via H&E staining (n = 19–46). Data is presented as mean ± SE. Bar graphs not sharing a common letter or symbol are significantly different from one another (P < 0.05). ME = Main Effect.

Because APN has been shown to be regulated by PPARγ, a master regular of adipogenesis, we assessed adipogenic markers including, PPARγ and PGC1-α^39,40^. In the gonadally-intact female mice, *PPARγ* was found to be increased in APN KO mice relative to WT littermates (Supplementary Fig. 6) (P < 0.05).

### APN deficiency dramatically reduces hepatic steatosis in male mice

We assessed the degree to which APN deficiency impacted hepatic steatosis (Fig. 7). When comparing between gonadally intact males and females, there was a main effect of genotype, with APN KO mice exhibiting less hepatic steatosis than WT littermates (P < 0.05). The post-hoc assessment showed that only male APN KO mice presented significantly less hepatic lipid accumulation than male WT mice, which was evident histologically (P < 0.05). Similarly, when comparing male and OVX mice, a main effect of genotype was found with APN KO mice exhibiting less hepatic steatosis (P < 0.05). However, no significant difference was found between WT OVX and APN OVX with respect to the degree of hepatic steatosis.

### Slight, but non-statistically significant differences in food intake and energy expenditure leading to a net difference in energy balance likely explain the reduced body weight exhibited by male APN KO mice

**Figure 8:**
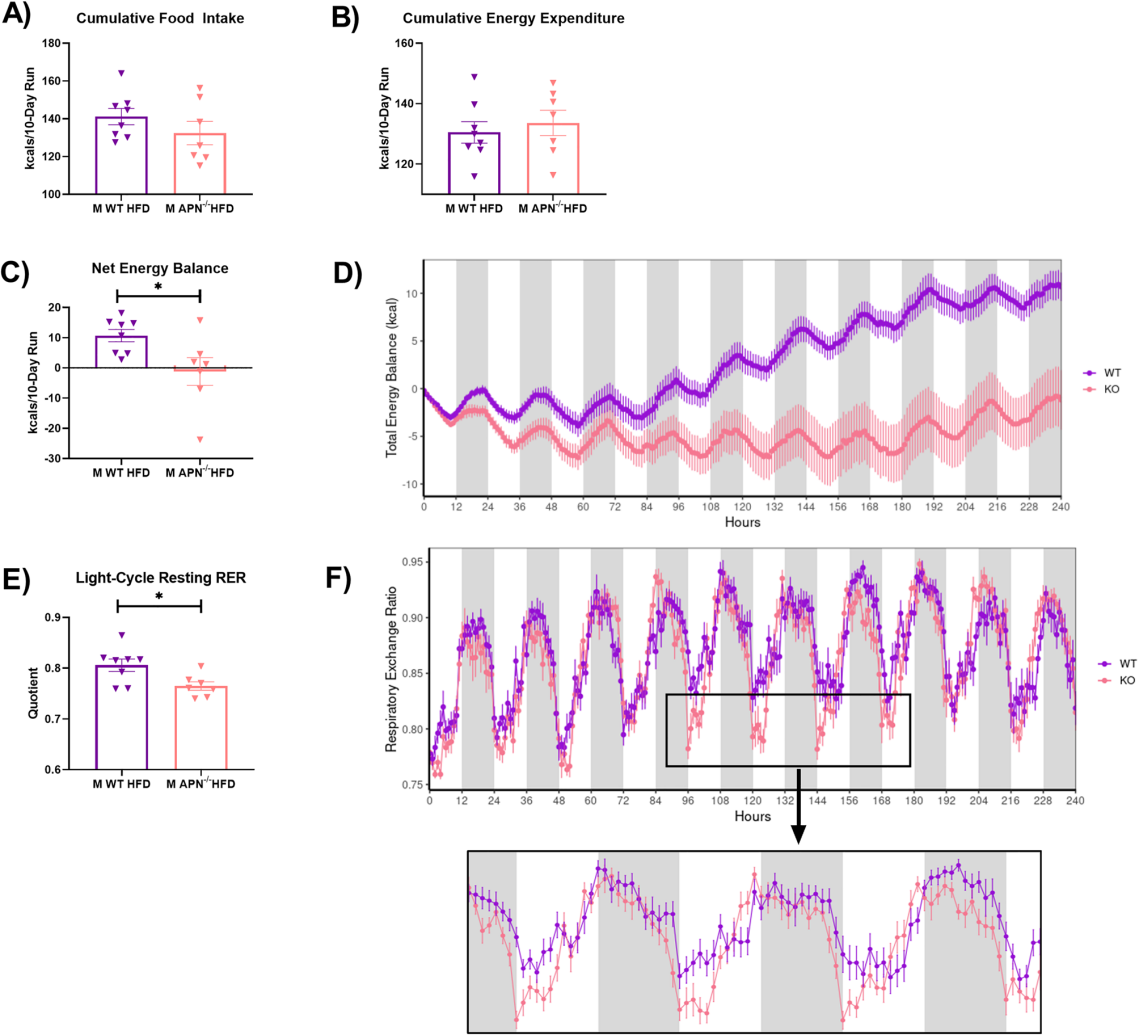
Slight, but non-statistically significant differences in food intake and energy expenditure leading to a net difference in energy balance likely explain the reduced body weight exhibited by male APN KO mice. Male APN^−/−^ and WT littermate controls (n = 7–8) were fed a HFD for 6 weeks and were placed into a 16-cage Promethion multichannel continuous measurement indirect calorimetry system (Sable System International, Las Vegas, NV, USA) on a 12-h light and 12-h dark cycle for a 11-day period. The data presented is (**A**) cumulative food intake, (**B**) cumulative energy expenditure, (**C**) net energy balance, (**D**) total energy balance over the course of the 10-day experiment assessed via CalR, (**E**) the RER calculated during the lowest 30-min period of energy expenditure during the light cycle, and (**F**) the RER over the course of the experimental run utilizing CalR. Data is presented as mean ± SE. *Signifies statistically significant differences (P < 0.05).

Although male and female APN KO mice presented a statistically significant difference in body weight gain, and visceral fat mass accumulation compared to their WT littermates, this phenotype was stronger in APN KO males relative to their male WT littermates. Given this more pronounced phenotype, we explored whether differences in food intake could explain these outcomes. Male APN KO (n = 10) and WT (n = 15) mice were singly housed for 16 weeks of dietary treatment (Supplementary Fig. 6). Similar to our initial experiment, we confirmed our previous finding that male APN KO mice do not gain as much body weight as their WT littermate controls (Supplementary Fig. 7A) (P < 0.05). Additionally, we found no difference in food intake over the course of the feeding period (Supplementary Fig. 6B), suggesting that differences in food intake do not explain the dissimilarities in body weight gain. Subsequently, we placed male APN KO and WT mice in high-resolution metabolic cages for a 10-day experiment. We did not find any statistically significant changes with respect to food intake (Fig. 8A), energy expenditure (Fig. 8B), physical activity, or resting energy expenditure between the two groups (Supplementary Table 1). However, when examining total energy balance, we found that the APN KO mice presented a significantly lower net energy balance than WT mice resulting in an ≈ 10 kcal difference (i.e. 7% of total energy intake) over the 10-day run between the two groups (Fig. 8C,D) (P < 0.05). This was paired with a lower RER (Supplementary Table 1) and lower resting RER as determined during the 30 min of lowest energy expenditure during the light cycle (Fig. 8E,F) (P < 0.05). Over time, this net energy balance deficit is likely responsible for the decreased body weight gain that the APN KO phenotype presents after approximately 12–14 weeks of HFD treatment. This outcome is explained by slight, non-statistical differences in food intake and energy expenditure.

## Discussion

It is well established that estrogen deficiency both in humans and animal models increases the susceptibility to obesity and metabolic dysfunction^4,41^. Our data corroborates this as estrogen deficiency (OVX) exacerbated HFD-induced obesity in female mice. However, here we show that despite a higher body fat percentage and a similar degree of hepatic and skeletal muscle lipid accumulation, female estrogen-deficient (OVX) HFD-fed mice exhibit significantly enhanced insulin sensitivity relative to HFD-fed males. Given that we found significant increases in plasma HMW APN in HFD OVX mice and contrary decreases in male HFD mice, we hypothesized that APN may be the key molecular factor regulating the enhanced insulin sensitivity exhibited by the estrogen-deficient females relative to the males. Utilizing APN KO mice we tested this hypothesis and found that APN deficiency limited adiposity gains, mitigated HFD-induced insulin resistance, and minimized adipose tissue inflammation in gonadally intact male and female mice, but not in OVX mice. Thus, APN is not the molecule that protects OVX HFD mice from the same degree of insulin resistance as HFD male mice. Moreover, there is an interaction between gonadal status and adiponectin tone; where basal APN negatively affects insulin action when mice are gonadally intact, but not in the setting of OVX.

From a clinical perspective, the data on APN as a therapeutic agent and the role it plays in metabolic disorders remains controversial and ambiguous. For example, a number of epidemiological studies have found that low circulating levels of APN have been linked to a variety of diseases as reviewed by Ye et al.^42^. However, other epidemiological data do not support a causal role of lower circulating APN levels in insulin resistance and T2D^43^. Furthermore, large-scale data have shown a positive association between circulating APN levels and mortality rate^8^, which has led to the suggestion that there exists an “APN Paradox” in which high levels of APN do not translate to beneficial metabolic outcomes in humans^44–46^. The mechanisms for this paradox are an ongoing field of investigation, but it is believed that there exists a state of “APN resistance”^47^. Indeed, APN resistance has been shown to precede skeletal muscle lipid accumulation and insulin resistance in HFD-fed rats^48^. Thus, our finding that circulating HMW APN is increased in HFD-fed gonadally intact and OVX females relative to LFD-fed females may be interpreted as a compensatory response to APN resistance. However, this same trend was not exhibited in male mice as HFD-fed male mice exhibited significantly less circulating HMW APN than LFD-fed males. Thus, more research is necessary to determine the effect that sex has on HMW APN formation and APN resistance in the setting of obesity.

Much of the molecular mechanisms responsible for APN action are based on basic science research. The vast majority of this research suggests a beneficial metabolic effect of APN action, which was recently highlighted in an elegant paper by Dr. Philipp Scherer’s group showing that APN “preserves metabolic fitness during aging”^10^. Nonetheless, when examining the literature closely, the discrepancies in the APN basic science literature with respect to APN deletion come to light.

Three different congenital knockout models (we have identified as the Kadowaki, Matsuzawa, and Scherer models) have found APN deletion to induce a phenotype of mild metabolic dysfunction with respect to insulin sensitivity and glucose tolerance^49–51^. Furthermore, Dr. Scherer’s group has recently shown that acute loss of APN elicits a more severe systemic insulin resistant phenotype and hyperlipidemia than the congenital KO model his group developed^52^. Similarly, overexpression of APN as well its receptors have been shown to provide substantial metabolic benefits^53,54^. Additionally, Dr. Gary Sweeney’s group has published a number of papers shown the metabolic benefits of APN action, particularly in skeletal muscle^55–58^. On the contrary, in a fourth congenital KO model (identified as the Chan model), APN deletion was found to increase skeletal muscle and hepatic beta oxidation without affecting glucose tolerance or insulin sensitivity^14^. The discrepancy in the Chan congenital KO model relative to the other three congenital models has been hypothesized to be attributed to the mechanism APN genetic disruption was achieved which is thought to lead to a gain-of-function, rather than a loss-of-function phenotype^52^. However, follow-up studies by different investigators utilizing either the Matsuzawa or Scherer models have uncovered phenotypes which differ than those described in the original publications. This suggests that environmental factors, rather than genetic factors, play a role in impacting the APN deficient phenotype. For example, although glucose metabolism was not assessed, utilizing the Matsuzawa model, Dr. Harvey Lodish’s group found that APN KO mice weigh less, exhibit significantly less epididymal fat weight, and show dramatically less hepatic steatosis when challenged with a high-fat diet (HFD)^13^—all findings not described in the original publication utilizing this mouse model^50^. Additionally, using the Scherer model it was found that these APN KO mice present reduced body weight and are resistant to HFD-induced obesity^12^—a phenotype also not described in the original publication of this model^51^.

Before initiating this study with the APN KO mouse model, we purposely reached out to Dr. Scherer in order to utilize the APN KO mice his group developed as we were aware of the controversy surrounding the different congenital APN KO models. Thus, in this study we utilized the Scherer APN KO model, generated littermate controls, used a significantly large sample size (n = 19–46), and verified non-detectable levels of plasma APN in all KO animals used. We found that APN deficiency dramatically reduced body weight gain and hepatic steatosis, particularly in males, which supports the finding of Dr. Harvey Lodish’s group^13^, and improves insulin sensitivity in gonadally intact males and females. It was our initial thought that perhaps the use of our unique custom diet (40% fat, 47% carbohydrate, and 13% protein of total kcals) was responsible for our findings. It should be noted that we have included a supplementary table (Supplementary Table 2) with this manuscript which details previous studies utilizing APN KO models in the setting of HFD-feeding and details the source and sex of the mouse model, the diet used in the investigation, outcomes with respect to body weight and adiposity changes, as well as any effects on inflammation and metabolism. The notion that our HFD may be responsible for our dissimilar results as other previous publications was based on the fact that the majority of previous studies utilizing congenital APN KO models have used diets consisting of > 45% of total kcals coming from fat, with the most common diet utilized being the 60% HFD from Research Diets. However, when reviewing the literature we found that Dr. Harvey Lodish’s group utilized the 60% HFD in their publication^13^, suggesting that our custom diet is not the primary factor regulating this phenotype as they found similar beneficial effects of APN deficiency on adiposity gains and hepatic steatosis.

In order to determine if a potential difference in food intake was responsible for the decreased adiposity displayed by the APN KO mice, we singly housed male WT and APN KO mice for a 16-week period. No differences in food intake were found. Subsequently, we placed male WT and APN KO mice in metabolic cages over a 10-day period after 6 weeks of HFD before there were statistically significant differences in body weight or body composition. Although we did not find and statistically significant differences with respect to energy expenditure or food intake, our instrumentation was sensitive enough to detect a clear difference in net energy balance and body weight between the two genotypes. A subtle decrease in food intake and a matching mild increase in energy expenditure resulted in a significantly lower net energy balance in the APN KO mice of approximately − 10 kcals (≈ 7% of total kcals) over the 10-day experiment. When examining the body weight graphs over time, it is evident that the male APN KO mice do not present a reduced body weight relative to the male WT mice until approximately 12–14 weeks of HFD. This suggests that the phenotype presented by the male APN KO mice is mild. Thus, it is likely that the lower net energy balance exhibited by the male APN KO mice, over time, explains the reduced body weight and decreased adiposity. The molecular mechanisms responsible for this mild, time-dependent phenotype are unclear.

An interesting finding of this investigation is that OVX negatively regulates the beneficial effects of APN deletion. Furthermore, we show that gonadally intact males benefit more from APN deficiency than do gonadally intact females. These findings suggest an interaction between gonadal status and APN tone, which we show impacts metabolic processes. The mechanism(s) responsible for this interaction remain poorly understood. Interestingly, we found PPARγ gene expression to be upregulated as a result of APN deficiency only in the gonadally intact female mice relative to WT littermates. Given the role that PPARγ has in regulating adipogenesis and its interaction with APN^39^, future studies should explore the impact that sex has on the PPARγ-APN crosstalk. A limitation of our investigation is that we did not include LFD groups in our APN KO experiment. Furthermore, we did not perform “add-back” experiments of globular APN or APN receptor agonists (AdipoRON). Thus, studies are needed to better understand the impact that sex, gonadal status, and diet have on the molecular effects of APN.

The initial impetus for utilizing the APN KO model was to try and explain the mechanism by which OVX HFD-females display enhanced insulin sensitivity relative to HFD males despite a similar degree of adiposity and ectopic lipid accumulation. We show that APN is not that mechanistic link. We can only surmise that although OVX HFD females and HFD males display a similar degree of ectopic lipid accumulation perhaps a difference in the content of lipid species known to induce insulin resistance (e.g. diacylglycerols or ceramides) in metabolic tissue is responsible for the dichotomy in insulin sensitivity between these groups^59,60^. Future studies would need to be designed to examine and test this hypothesis.

In conclusion, we find that APN ablation does not have an effect on obesity development, glucose metabolism, or insulin resistance in OVX females. On the contrary, APN deficiency in gonadally intact mice mitigates obesity development and positively influences insulin sensitivity in a HFD setting, which is likely explained by subtle changes in food intake and energy expenditure leading to a lower net energy balance over time. Whether these results are due to compensatory factors as a result of congenital deletion of APN is unknown. Given the inconsistent findings with respect to APN deficiency in the hands of different investigators paired with the fact that we show that sex as well as gonadal status impact the APN deficient phenotype, future studies are needed to tease out the environmental and physiological (e.g. sex steroids) factors which are responsible for these effects.

## Supplementary Information

Supplementary Information.

## Data Availability

All data is available upon request to the corresponding author.

## Abbreviations

APN: Adiponectin
HFD: High-fat diet
LFD: Low-fat diet
OVX: Ovariectomized
T2D: Type-2 diabetes
WT: Wildtype
