# Supplementary material for: Congenital adiponectin deficiency mitigates high-fat-diet-induced obesity in gonadally intact male and female, but not in ovariectomized mice
Source: Sci Rep. 2022 Oct 5;12:16668. doi: 10.1038/s41598-022-21228-x (PMC9534911; doi:10.1038/s41598-022-21228-x)
Supplement: Supplementary file 1 — Supplementary Information. [file 41598_2022_21228_MOESM1_ESM.docx]

**Title: Congenital adiponectin deficiency mitigates high-fat-diet-induced obesity in gonadally intact male and female, but not in ovariectomized mice**

**Authors:** Christian A. Unger^1^, Ahmed K. Aladhami^1,2^, Marion C Hope III^1^, Sahar Pourhoseini^1^, Mitzi Nagarkatti^1^, Owen P. McGuinness^3^, E. Angela Murphy^1^, Kandy T. Velázquez^1^, and Reilly T. Enos^1^

**Affiliation:** ^1^University of South Carolina-School of Medicine, Columbia, SC, Department of Pathology, Microbiology, and Immunology.

^2^ University of Baghdad, Nursing College, Baghdad, Iraq

^3^ Department of Molecular Physiology and Biophysics, Vanderbilt University School of Medicine, Nashville, TN 37232, USA

**Address for Correspondence:** Reilly Enos. Department of Pathology, Microbiology and Immunology. University of South Carolina School of Medicine 6439 Garners Ferry Rd Columbia, SC 29209; Tel. #(803)216-3414; Fax. (803)216-3413; email: [enosr@email.sc.edu](mailto:enosr@email.sc.edu)


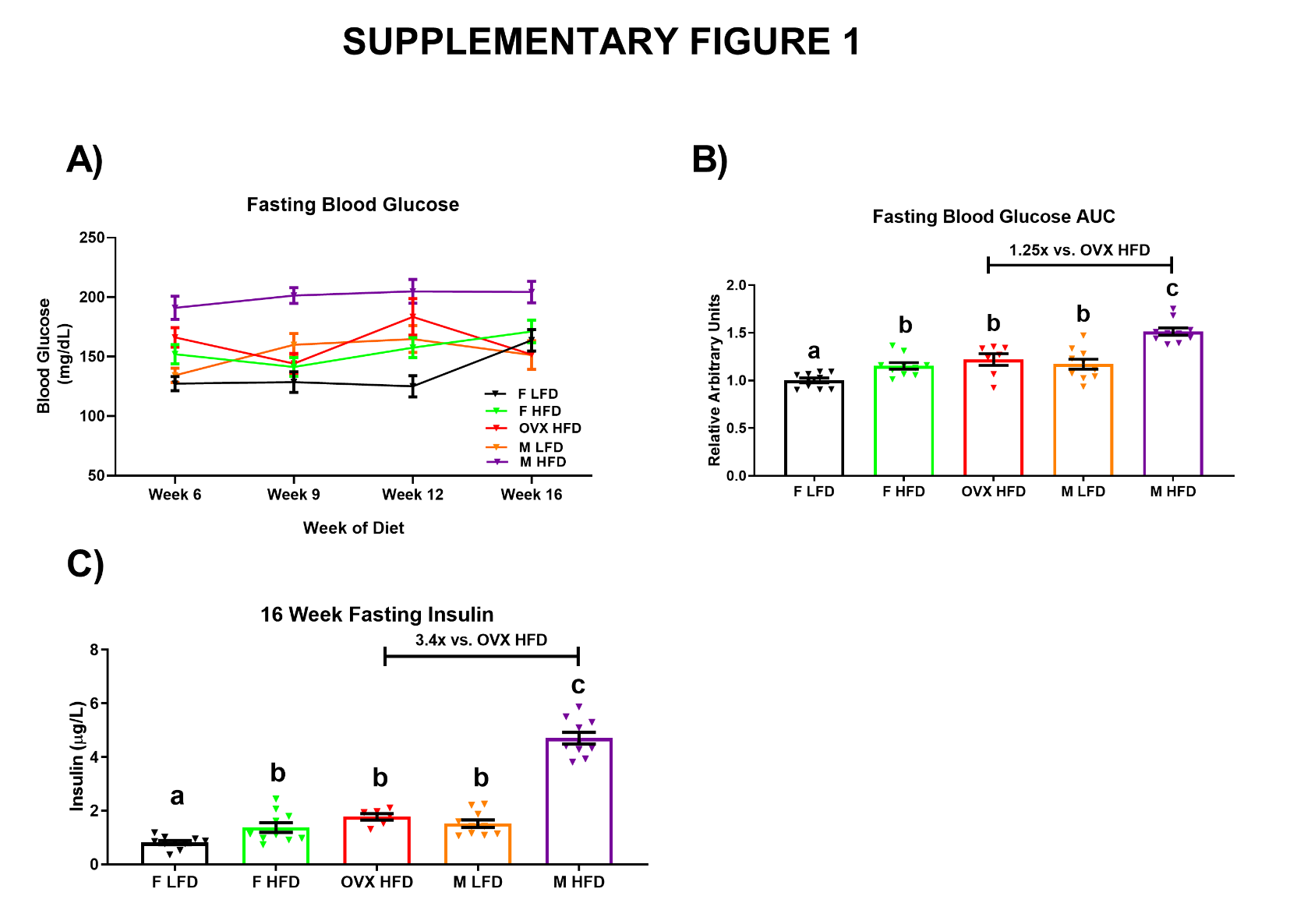


**Supplementary Figure 1. Despite a similar degree of adiposity, HFD estrogen-deficient females exhibit lower circulating fasting blood glucose and insulin levels.** Over the course of 16-weeks of either LFD or HFD consumption (n=7-10), A) fasting blood glucose levels were measured incrementally over the course of the study, and B) the area under the curve (AUC) was calculated. After 16 weeks of diet, C) fasting plasma insulin levels were assessed. Data is presented as mean ± SE. Bar graphs not sharing a common letter are significantly different from one another (P<0.05).

**
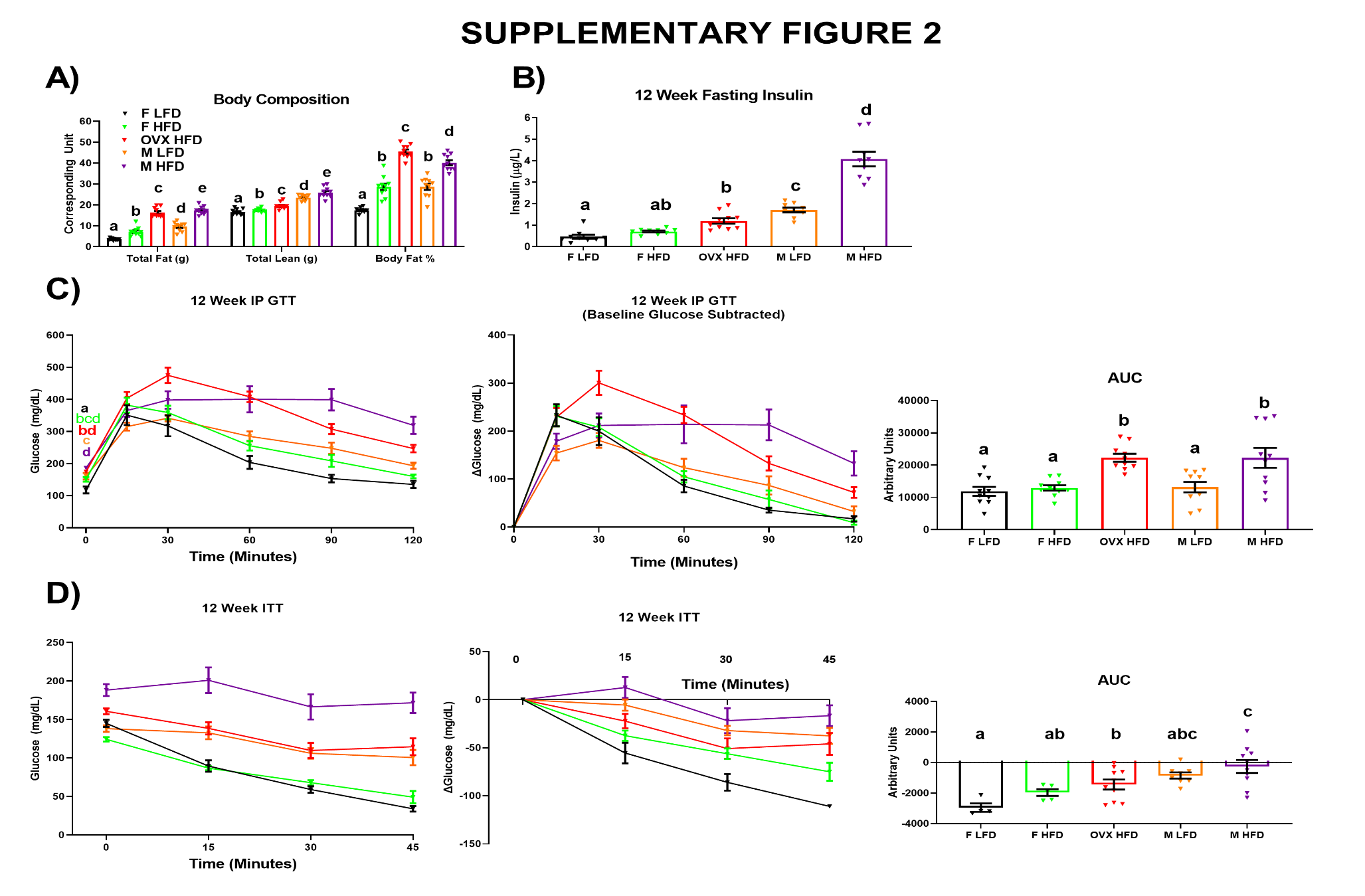
Supplementary Figure 2. Despite a higher body fat%, HFD estrogen-deficient females exhibit enhanced insulin sensitivity relative to HFD males after 12 weeks of HFD.** After 12 weeks of LFD or HFD, mice (n=10) were assessed for A) body composition and metabolically by examining B) fasting insulin levels, C) a glucose tolerance test (GTT), D) insulin tolerance test (ITT). Data is presented as mean ± SE. Bar graphs not sharing a common letter are significantly different from one another (P<0.05).

**
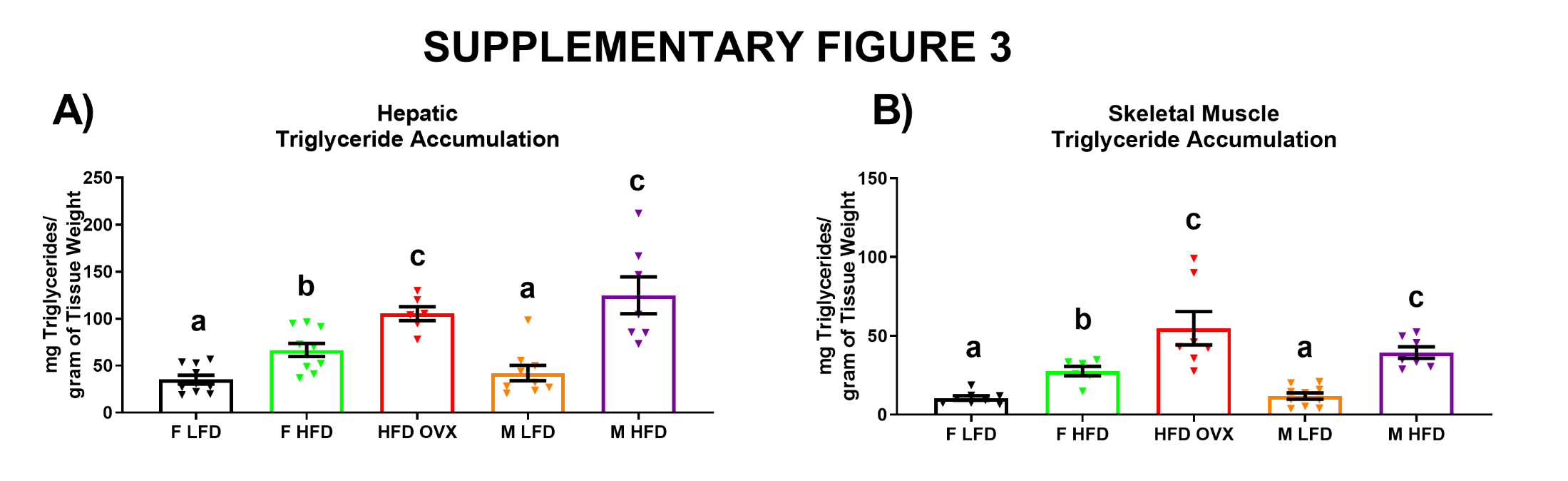
Supplementary Figure 3. HFD estrogen-deficient females and HFD males have similar levels of ectopic lipid accumulation.** After 16 weeks of LFD or HFD, the A) liver and B) skeletal muscle (plantaris) mice (n=7-10) were assessed for triglyceride accumulation. Data is presented as mean ± SE. Bar graphs not sharing a common letter are significantly different from one another (P<0.05).


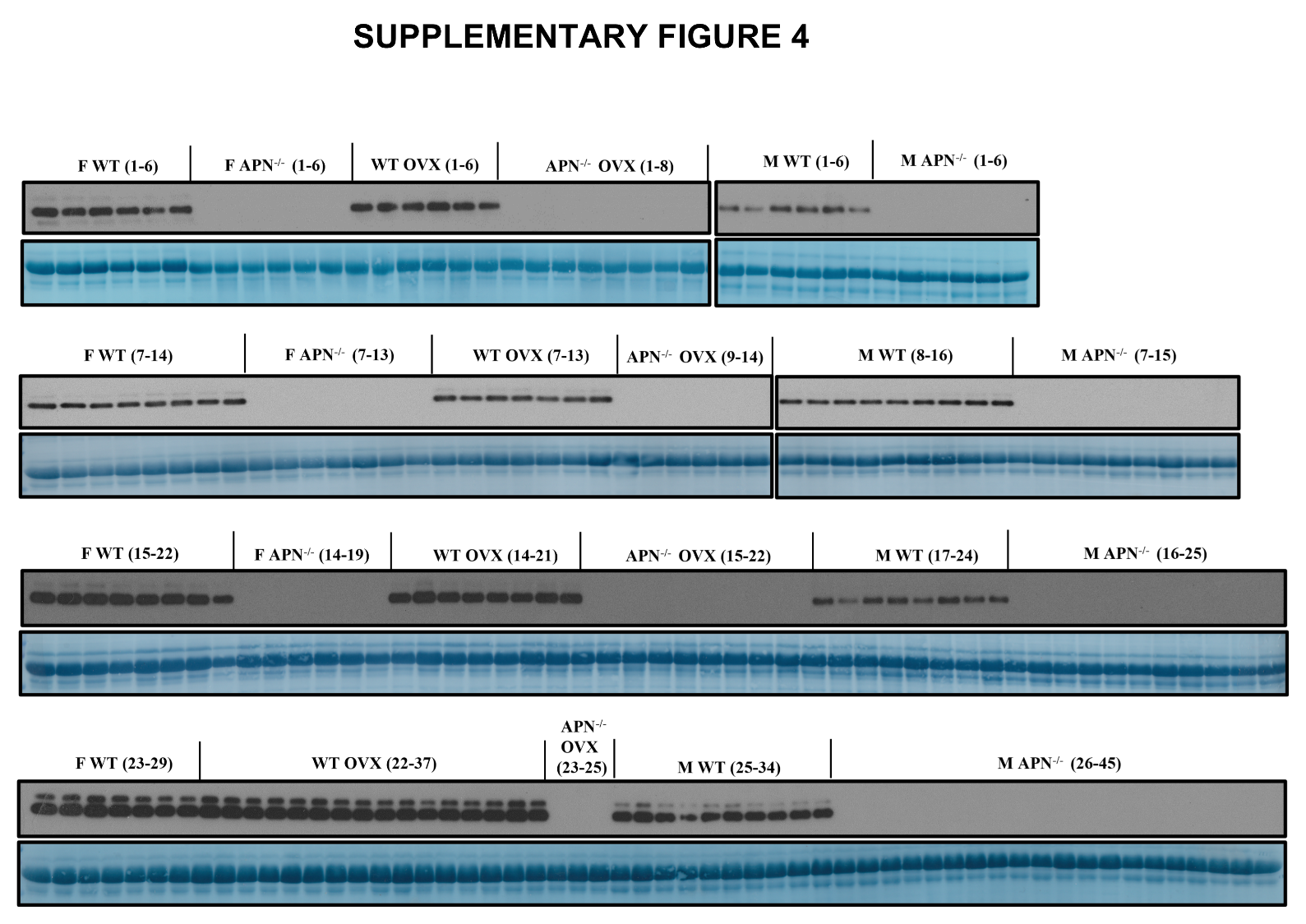


**Supplementary Figure 4. APN KO mice do not have any circulating plasma APN.** The plasma of all experimental mice in the APN KO study was assessed for circulating APN.


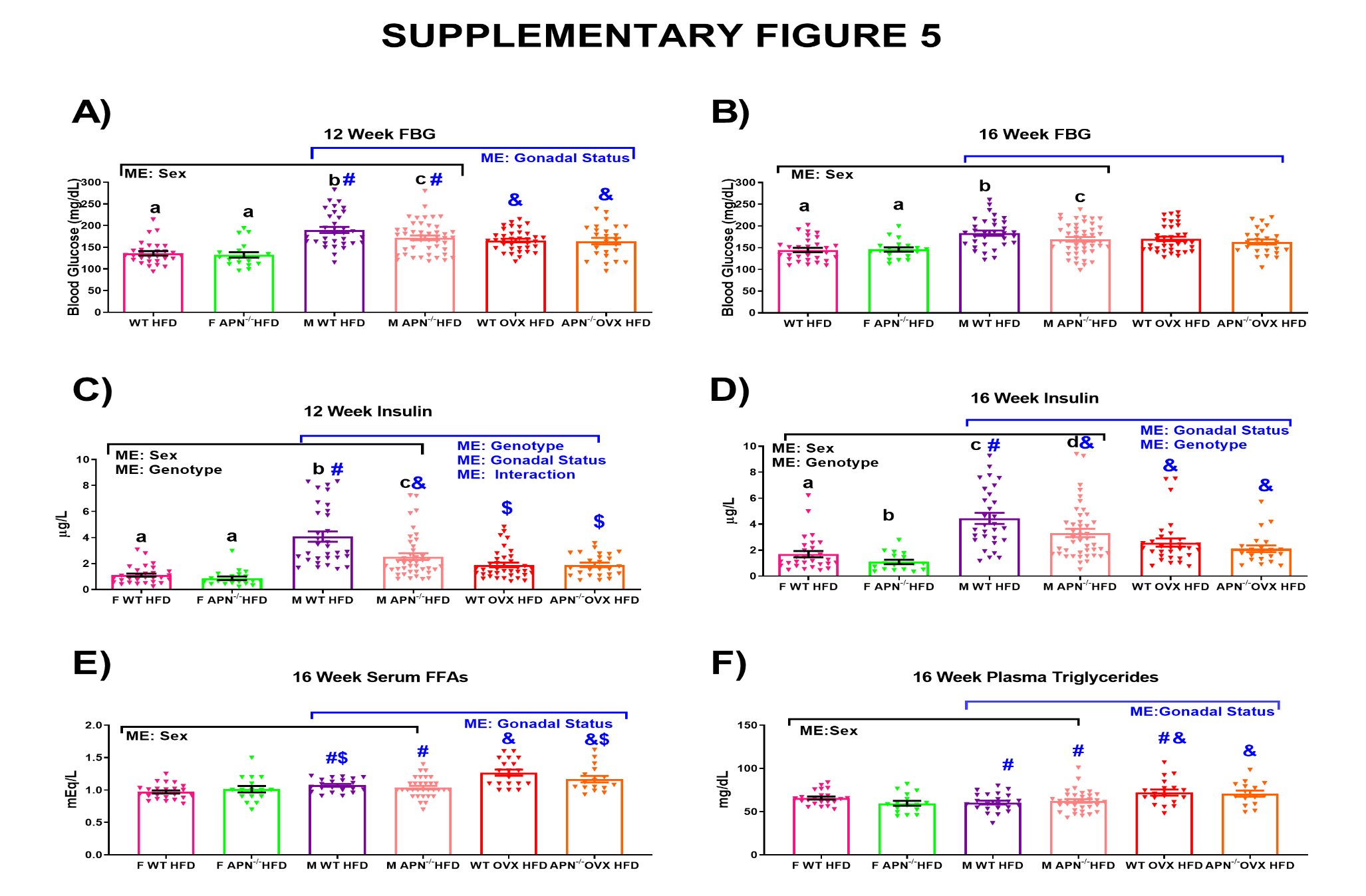


**Supplementary Figure 5. Fasting blood glucose and insulin are affected by APN deficiency in sexually intact males and females, but not ovariectomized females.** After 12 and 16 weeks of HFD, A-B) fasting blood glucose, C-D) fasting insulin, E) fasting serum free-fatty acids (FFAs), and F) plasma triglycerides were assessed in male and female (both intact and OVX) APN deficient mice (M and F APN^-/-^) and wildtype (WT) littermate controls (n=19-46). Data is presented as mean ± SE. Bar graphs not sharing a common letter or symbol are significantly different from one another (P<0.05). ME = Main Effect.

**
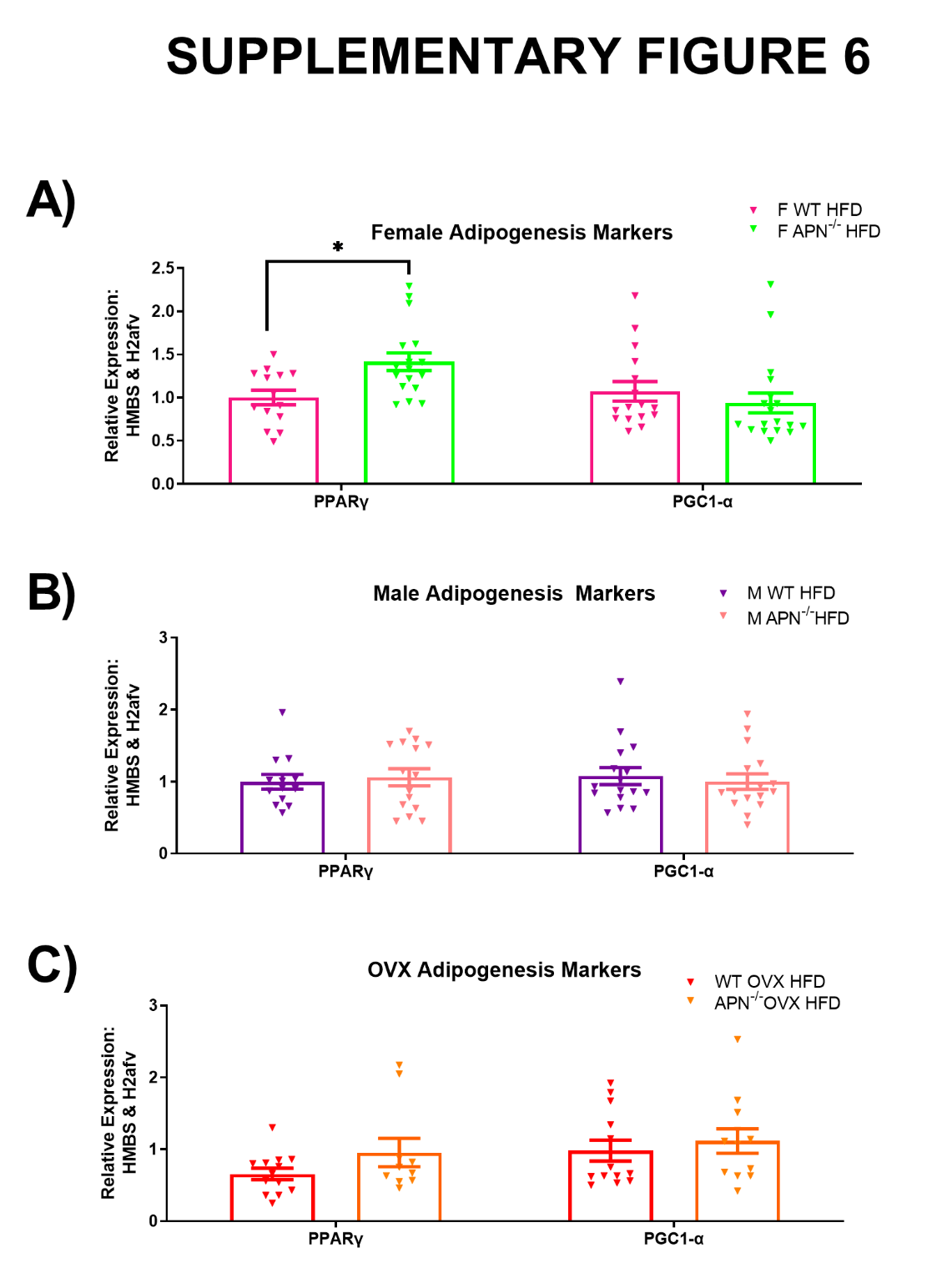
Supplementary Figure 6. Markers of Adipogenesis.** After 16 weeks of HFD, adipose tissue markers of adipogenesis were assessed via qRT-PCR in A) F WT and APN^-/-^ mice (n=15-16)**,** B) M WT and APN^-/-^ mice (n=15-16), and C) OVX WT and APN^-/-^ mice (n=10-13). Data is presented as mean ± SE. *Signifies statistically significant differences (P<0.05).


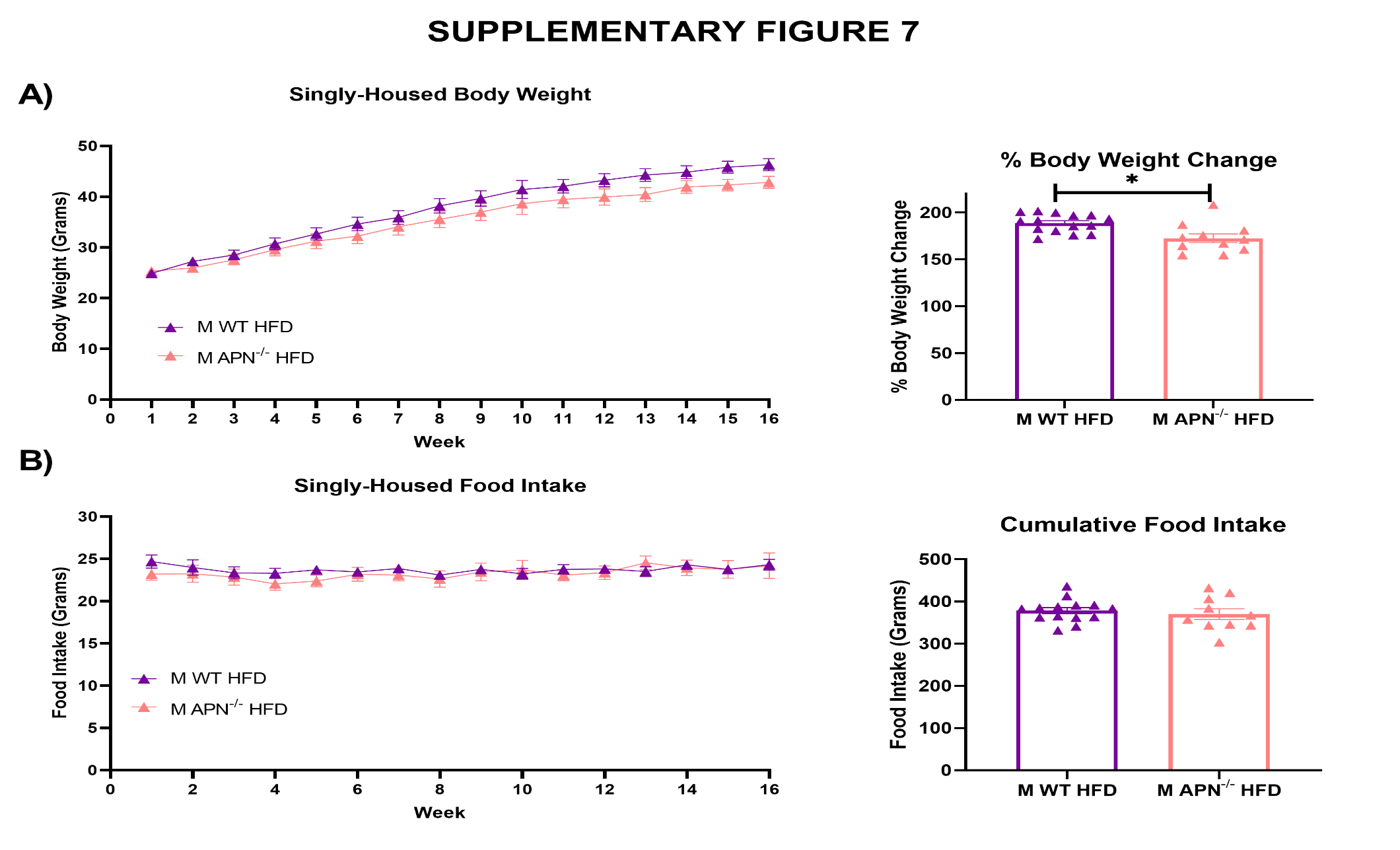


**Supplementary Figure 7. There is no difference in food intake between male WT and APN KO mice.** Male WT and APN KO (n=10-15) mice were singly housed and fed a HFD for 16 weeks. A) Body weight and % body weight change, as well as B) food intake was assessed over the course of the experiment. Data is presented as mean ± SE. *Signifies statistically significant differences (P<0.05).

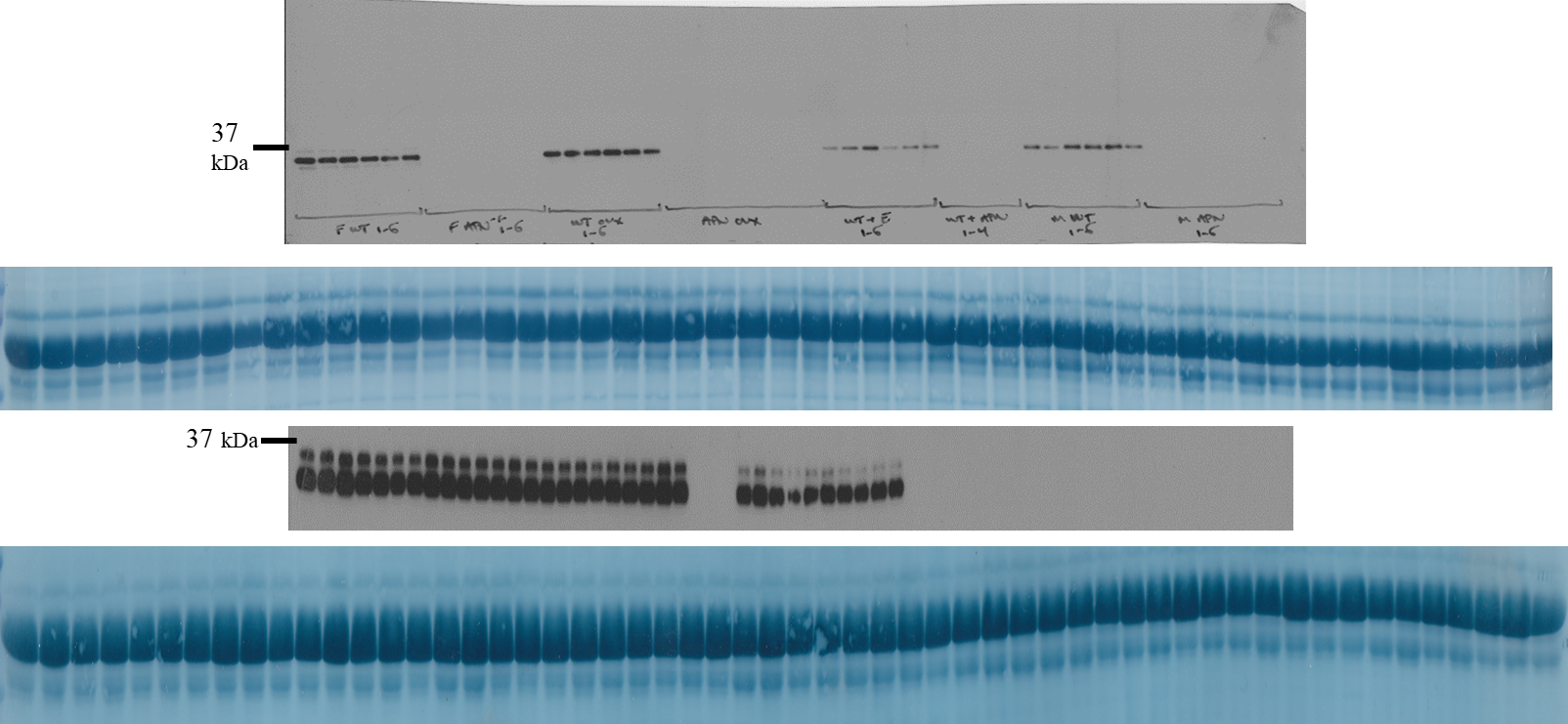


| ***Supplementary Table 1.* Indirect Calorimetry Data** | | | | | | | |
| --- | --- | --- | --- | --- | --- | --- | --- |
| **Avg. Temp Celsius** | **Unadjusted** |  |  |  | **ANCOVA Adjusted** |  |  |
| **22.50 (.102)** | **WT** | **APN** | **P-Value** | **WT** | **APN** | **P-Value** |  |
| **Total EE (kcal/day)** | 13.05 (0.23) | 13.36 (0.246) | 0.3674 | 12.97 (0.23) | 13.45 (0.246) | 0.1872 |  |
| **Energy Intake (kcal/day)** | 14.12 (0.458) | 13.24 (0.489) | 0.2175 | 14.05 (0.459) | 13.31 (0.491) | 0.2919 |  |
| **Resting EE (kcal/hr)** | 0.44 (0.01) | 0.45 (0.011) | 0.5999 | 0.43 (0.01) | 0.45 (0.011) | 0.3441 |  |
| **Non-resting EE (kcal/day)** | 2.54 (0.138) | 2.63 (0.148) | 0.6523 | 2.54 (0.138) | 2.63 (0.148) | 0.6692 |  |
| **O2 Consumption (Light Cycle Avg)** | 1.75 (0.035) | 1.78 (0.037) | 0.5454 | 1.73 (0.035) | 1.79 (0.037) | 0.2843 |  |
| **Co2 Production (Light Cycle Avg)** | 1.46 (0.034) | 1.46 (0.036) | 0.8612 | 1.45 (0.034) | 1.48 (0.037) | 0.5411 |  |
| **Avg RER (Light Cycle Avg)** | 0.906 (.0117) | 0.837 (.0085) | 0.000747495 |  |  |  |  |
| **All Meters (day)** | 172.3 (12.55) | 190.9 (24.23) | 0.547193486 |  |  |  |  |

**Supplementary Table 1. Indirect calorimetry data.** Male APN^-/-^ and WT littermate controls (n=7-8) were fed a HFD for 6 weeks and were placed into a 16-cage Promethion multichannel continuous measurement indirect calorimetry system (Sable System International, Las Vegas, NV, USA) on a 12-h light and 12-h dark cycle for a 11-day period.

|  | | | | | | | | | |
| --- | --- | --- | --- | --- | --- | --- | --- | --- | --- |
| *Supplementary Table 2. Congenital APN^-/-^ Studies* | | | | | | | | | |
| **Authors & APN KO Model Source** | **Strain & Sex** | **Littermates** | **Diet & Duration** | **Outcomes comparing APN^-/-^ HFD vs. WT HFD** | | | | | |
|  |  |  |  | **Body Weight** | **Adiposity** | **Inflammation** | **Glucose Metabolism** | **Lipid Profile** | **NAFLD** |
| Qiao *et al.^1^*  (Scherer Model) | C57BL/6 Strain  (Male) | Yes | 60% HFD for 8 Weeks | ↓ | ↓ | NA | IR: ↑ (Data Not Shown) | FFA: ↑ | NA |
| Guo *et al.^2^*  (Chan Model) | C57BL/6 Strain  (Male) | Yes | 45% HFD for 22 Weeks | ↑ | ↑ | NA | AUC GTT: ↑ | TAG: ↑  TC: NA  HDL-C: NA  LDL-C: NA  FFA: NA | NA |
| Liu *et al*. ^3^  (Matsuzawa Model) | C57BL/6 Strain  (Male & Female) | Not Specified | 60% HFD for 30 days | ↓ for both sexes | ↓ for both sexes | NA | NA | NA | Hepatic Steatosis: ↓ both sexes |
| Maeda *et al*.^4^  (Matsuzawa Model) | C57BL/6 Mice  (Male) | Yes | Described as High-Sucrose/High-Fat, yet is the AIN-93G diet (15.8% Fat, 64% Carbohydrate) for 2 Weeks | No Change | No Change | Plasma TNF-α: ↑ | FBG: ↑  Fasting Insulin: ↑  HOMA-IR: ↑  ITT AUC: ↑ | TAG: ↑  TC: NA  HDL-C: NA  LDL-C: NA  FFA: ↑ | Hepatic Triglyceride: No Change |
| Aprahamian *et al.* ^5^  (Matsuzawa Model) | C57BL/6 Mice  (Male) | Yes | 60% HFD for 32 Weeks | No Change | ↓ | Adipose Tissue  Crown-Like Structures: No Change | FBG: No Change  Fasting Insulin: No Change  ITT AUC: No Change | TAG: NA  TC: NA  HDL-C: NA  LDL-C: NA  FFA: No Change | Not Assessed Statistically |
| Ma *et al. ^6^*  (Chan Model) | C57BL/6 Mice  (Male & Female) | Yes | High-Fat/High-Fructose for 7 months | No Change | No Change | NA | FBG: No Change  Fasting Insulin: No Change  GTT: No Change  ITT: No Change  Hyperinsulinemic-Euglycemic Clamp: No Change | NA with HF/HS Diet | Hepatic and Muscle β-Oxidation: ↑ |
| Lee *et al.^7^*  (Model Not Specified) | C57BL/6 Mice  (Male) | Not Specified | 45% HFD for 8-12 Weeks | No Change | ↑ | NA | FBG: ↑  GTT: Worse | TAG: ↑  TC: ↑  HDL-C: NA  LDL-C: NA  FFA: ↑ | NA |
| Hecker *et al.^8^*  (Matsuzawa Model) | C57BL/6 Mice  (Male) | No | 45% HFD for 8 Weeks | No Change | ↓ | NA | FBG: No Change  Fasting Insulin: No Change | TAG: No Change  TC: NA  HDL-C: NA  LDL-C: NA  FFA: No Change | NA |
| Nawrocki *et al.^9^*  (Scherer Model) | C57BL/6 Mice  (Male) | Yes | 60% HFD for 10 Weeks | No Change | NA | NA | GTT: Worse  Fasting Insulin: No Change | NA | NA |
| Kubota *et al. ^10^*  (Kadowaki Model) | C57BL/6 Mice  (Male) | Yes | 32% Fat (w/w) for 10 Weeks | No Change | NA | NA | GTT: Worse  ITT: Impaired | NA | NA |
| Asano *et al.* ^11^  (Kadowaki Model) | C57BL/6 Mice  (Male) | Not Specified | 60% HFD for 24 & 48 Weeks | No Change | NA | Hepatic  TNF-α mRNa: ↑ (24 Week only) | NA | TAG: No Change  TC: No Change  HDL-C: NA  LDL-C: NA  FFA: NA | ALT: No Change  Hepatic TAG: No Change  Fibrosis: ↑ (48 Week only) |
| Liu *et al.* ^12^  (Matsuzawa Model) | C57BL/6 Mice  (Male) | Yes | 60% HFD for 2, 4, and 6 Weeks | NA | NA | NA | FBG: No Change  Fasting Insulin: No Change  IP-GTT: Worse  HOMA-IR: Worse | NA | NA |
| Liu *et. al.^13^*  (Matsuzawa Model) | C57BL/6 Mice  (Male) | Yes | 60% HFD for 6 Weeks | NA | NA | NA | FBG: No Change  IP-GTT: Worse  Glucose Infusion Rate: ↓ | FFA: ↑ | NA |
| Perieira *et. al.^14^*  (Chan Model) | C57BL/6 Mice  (Male) | Not Specified | 59% HFD for 16 Weeks | No Change | No Change | Kidney  Nephrin: ↓  Albuminuria: ↑ | IPGTT: No Change | TAG: No Change | NA |
| Guo *et. al.^15^*  (Chan Model) | C57BL/6 Mice  (Male) | Not Specified | 45% HFD for 20 Weeks | ↑ | NA | Liver:  Cleaved Caspase-3: ↓  Bax: ↓  p-STAT3: ↓  p-JNK: ↑  p-Iκβ: ↓ | NA | NA | ALT: ↓  AST: No Change  AST/ALT Ratio: ↑  Steatosis: No Change |
| Mendonca *et al.^16^*  (Chan Model) | C57BL/6 Mice  (Male) | Not Specified | 59% HFD for 8 Weeks | ↓ | ↓ | Liver:  SCD1: ↓ | IP-GTT: Worse  ITT: Worse | NA | Steatosis: ↑ |
| Li *et. al.^17^*  (Scherer Model) | C57BL/6 Mice  (Male) | Yes | 60% HFD 36, 50, & 102 Weeks | NA | NA | Adipose Tissue:  ↑  Liver:  ↑  Kidney:  ↑ | OGTT: Worse | TG Clearance: Worse | ALT: ↑  AST: ↑  Fibrosis: ↑ |
| **Data Summary** | **Body Weight ∆** | | **Adiposity ∆** | | **Glucose Metabolism** | |  |  |  |
|  | ↔ | 9/14 = 65% | ↔ | 3/10 = 30% | ↔ | 4/14 = 29% |  |  |  |
|  | ↓ | 3/14 = 21% | ↓ | 5/10 = 50% | ↓ | 10/14 = 71% |  |  |  |
|  | ↑ | 2/28 = 14% | ↑ | 2/10 = 20% | ↑ | 0/14 = 0% |  |  |  |

**Supplementary Table 2. Details of papers utilizing various congenital APN KO mouse models**. Data summary includes only those studies that assessed the given outcome. ↔ = No Change, ↓ = Decrease, ↑ = Increase, NA = Not Assessed, FBG = Fasting Blood Glucose, TAG = Triglycerides, TC = Total Cholesterol, HDL-C = High-Density Lipoprotein Cholesterol, LDL-C = Low-Density Lipoprotein Cholesterol, FFA = Free-Fatty Acids.

**REFERENCES**

1. Qiao L, Kinney B, Schaack J, Shao J. Adiponectin Inhibits Lipolysis in Mouse Adipocytes. Diabetes 2011;60:1519-27.

2. Guo R, Zhang Y, Turdi S, Ren J. Adiponectin knockout accentuates high fat diet-induced obesity and cardiac dysfunction: Role of autophagy. Biochimica et Biophysica Acta (BBA) - Molecular Basis of Disease 2013;1832:1136-48.

3. Liu Q, Yuan B, Lo K, Patterson H, Sun Y, Lodish HF. Adiponectin regulates expression of hepatic genes critical for glucose and lipid metabolism. Proceedings of the National Academy of Sciences 2012;109:14568-73.

4. Maeda N, Shimomura I, Kishida K, et al. Diet-induced insulin resistance in mice lacking adiponectin/ACRP30. Nature Medicine 2002;8:731-7.

5. Aprahamian TR. Elevated adiponectin expression promotes adipose tissue vascularity under conditions of diet-induced obesity. Metabolism 2013;62:1730-8.

6. Ma K, Cabrero A, Saha PK, et al. Increased β-Oxidation but No Insulin Resistance or Glucose Intolerance in Mice Lacking Adiponectin. Journal of Biological Chemistry 2002;277:34658-61.

7. Lee EB, Warmann G, Dhir R, Ahima RS. Metabolic Dysfunction Associated with Adiponectin Deficiency Enhances Kainic Acid-Induced Seizure Severity. The Journal of Neuroscience 2011;31:14361-6.

8. Hecker PA, O'Shea KM, Galvao TF, Brown BH, Stanley WC. Role of adiponectin in the development of high fat diet-induced metabolic abnormalities in mice. Hormone and metabolic research = Hormon- und Stoffwechselforschung = Hormones et métabolisme 2010;43:100-5.

9. Nawrocki AR, Rajala MW, Tomas E, et al. Mice Lacking Adiponectin Show Decreased Hepatic Insulin Sensitivity and Reduced Responsiveness to Peroxisome Proliferator-activated Receptor γ Agonists. Journal of Biological Chemistry 2006;281:2654-60.

10. Kubota N, Terauchi Y, Yamauchi T, et al. Disruption of adiponectin causes insulin resistance and neointimal formation. Journal of Biological Chemistry 2002;277:25863-6.

11. Asano T, Watanabe K, Kubota N, et al. Adiponectin knockout mice on high fat diet develop fibrosing steatohepatitis. Journal of gastroenterology and hepatology 2009;24:1669-76.

12. Liu Y, Palanivel R, Rai E, et al. Adiponectin stimulates autophagy and reduces oxidative stress to enhance insulin sensitivity during high-fat diet feeding in mice. Diabetes 2015;64:36-48.

13. Liu Y, Turdi S, Park T, et al. Adiponectin corrects high-fat diet-induced disturbances in muscle metabolomic profile and whole-body glucose homeostasis. Diabetes 2013;62:743-52.

14. Pereira BMV, Thieme K, de Araujo L, Rodrigues AC. Lack of adiponectin in mice accelerates high-fat diet-induced progression of chronic kidney disease. Life Sci 2020;257:118061.

15. Guo R, Nair S, Zhang Y, Ren J. Adiponectin deficiency rescues high-fat diet-induced hepatic injury, apoptosis and autophagy loss despite persistent steatosis. Int J Obes (Lond) 2017;41:1403-12.

16. de Mendonca M, Dos Santos BAC, de Sousa E, Rodrigues AC. Adiponectin is required for pioglitazone-induced improvements in hepatic steatosis in mice fed a high-fat diet. Mol Cell Endocrinol 2019;493:110480.

17. Li N, Zhao S, Zhang Z, et al. Adiponectin preserves metabolic fitness during aging. Elife 2021;10.
